# Design, synthesis, and biological evaluation of novel halogenated chlorido[*N,N*′-bis(salicylidene)-1,2-bis(3-methoxyphenyl)ethylenediamine]iron(III) complexes as anticancer agents

**DOI:** 10.1007/s00775-024-02067-9

**Published:** 2024-08-12

**Authors:** Astrid Dagmar Bernkop-Schnürch, Klaus Huber, Armida Clauser, Monika Cziferszky, Daniel Leitner, Heribert Talasz, Martin Hermann, Stephan Hohloch, Ronald Gust, Brigitte Kircher

**Affiliations:** 1 Department of Pharmaceutical Chemistry, Institute of Pharmacy, CMBI—Center for Molecular Biosciences Innsbruck, CCB—Center for Chemistry and Biomedicine, University of Innsbruck, Innrain 80-82, 6020 Innsbruck, Austria; 2 Immunobiology and Stem Cell Laboratory, Department of Internal Medicine V (Hematology and Oncology), Medical University of Innsbruck, Anichstraße 35, 6020 Innsbruck, Austria; 3 Department of General, Inorganic and Theoretical Chemistry, University of Innsbruck, Innrain 80-82, 6020 Innsbruck, Austria; 4 Biocenter, Institute of Medical Biochemistry, Protein Core Facility, Medical University of Innsbruck, Innrain 80-82, 6020 Innsbruck, Austria; 5 Department of Anesthesiology and Critical Care Medicine, Medical University of Innsbruck, Anichstraße 35, 6020 Innsbruck, Austria; 6 Tyrolean Cancer Research Institute, Innrain 66, 6020 Innsbruck, Austria

**Keywords:** Iron(iii) salene, Stability, Lipophilicity, Cellular uptake, Anticancer drugs

## Abstract

Iron(III) complexes based on *N,N*´-bis(salicylidene)ethylenediamine (salene) scaffolds have demonstrated promising anticancer features like induction of ferroptosis, an iron dependent cell death. Since poor cellular uptake limits their therapeutical potential, this study aimed to enhance the lipophilic character of chlorido[*N,N*′-bis(salicylidene)-1,2-bis(3-methoxyphenyl)ethylenediamine]iron(III) complexes by introducing lipophilicity improving ligands such as fluorine (**X1**), chlorine (**X2**) and bromine (**X3**) in 5-position in the salicylidene moieties. After detailed characterization the binding to nucleophiles, logP values and cellular uptake were determined. The complexes were further evaluated regarding their biological activity on MDA-MB 231 mammary carcinoma, the non-tumorous SV-80 fibroblast, HS-5 stroma and MCF-10A mammary gland cell lines. Stability of the complexes in aqueous and biological environments was proven by the lack of interactions with amino acids and glutathione. Cellular uptake was positively correlated with the logP values, indicating that higher lipophilicity enhanced cellular uptake. The complexes induced strong antiproliferative and antimetabolic effects on MDA-MB 231 cells, but were inactive on all non-malignant cells tested. Generation of mitochondrial reactive oxygen species, increase of lipid peroxidation and induction of both ferroptosis and necroptosis were identified as mechanisms of action. In conclusion, halogenation of chlorido[*N,N*′-bis(salicylidene)-1,2-bis(3-methoxyphenyl)ethylenediamine]iron(III) complexes raises their lipophilic character resulting in improved cellular uptake.

**Graphical abstract:**

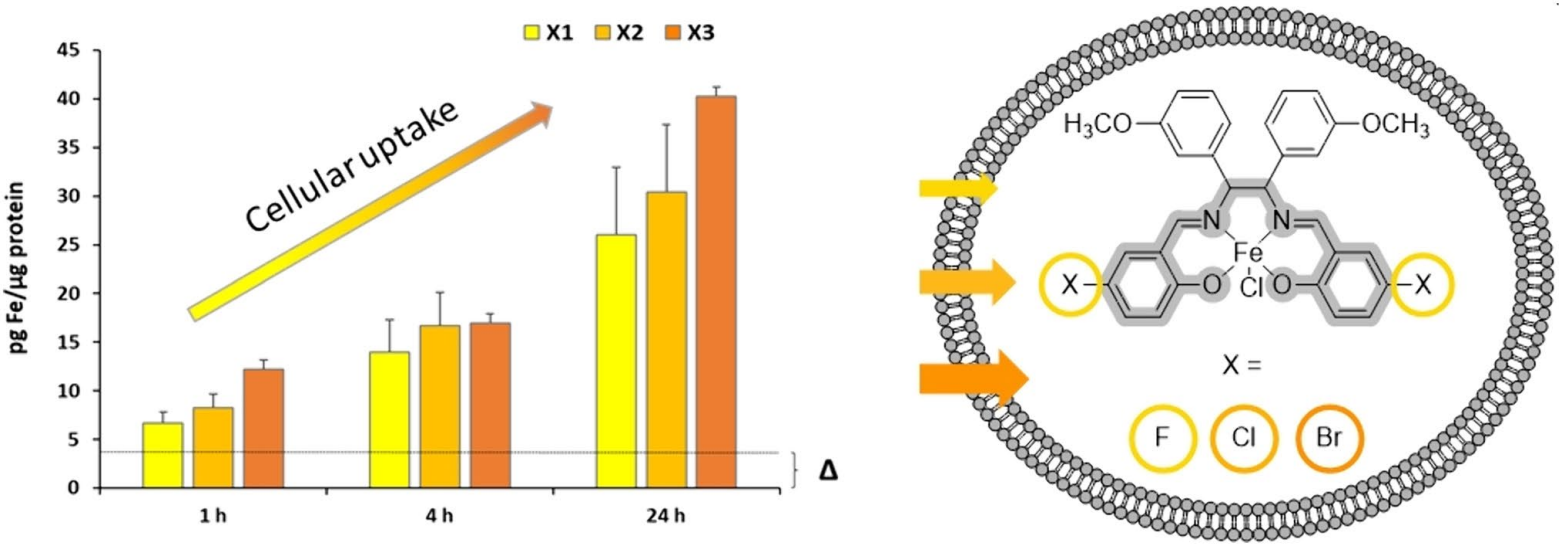

**Supplementary Information:**

The online version contains supplementary material available at 10.1007/s00775-024-02067-9.

## Introduction

Metal salene (*N,N*′-bis(salicylidene)ethylenediamine) complexes are a class of coordination compounds, which have gained significant attention in the field of inorganic chemistry due to their unique structures and versatile properties [1]. The term "salene" refers to the Schiff base ligands derived from the condensation of a diamine and a salicylaldehyde. These ligands can form stable complexes with various metal ions. Therefore, salene complexes with various metal ions e.g., Mn^3+^ [2], Ni^2+^ [3], Cr^3+^ [4], Co^2+/3+^ [3, 5, 6], Ru^3+^ [7] or Fe^3+^ [8, 9] were reported as potential anticancer drugs.

Breast cancer is a complex and potentially life-threatening disease that primarily affects women [10], but can also occur in men [11]. It is one of the most common cancers worldwide and a significant cause of morbidity and mortality [10, 12]. Thus, the development of anticancer drugs targeting and disrupting the mechanisms that drive cancer-cell growth and spread, plays a crucial role in the fight against this disease [13].

Ferroptosis [14–17], a form of non-apoptotic cell death coined in 2012, can be regarded as a promising mode of action for cancer treatment, including breast cancer [18–21]. It was originally described as a unique form of cell death induced by small molecules such as Erastin and RSL3, which inhibit the cysteine/glutamate antiporter (xc- system) or block glutathione peroxidase 4 (GPx4) [14, 22]. Both types of inhibitors exhibit antibreast cancer activity [16, 22, 23].

Since ferroptosis is characterized by iron induced lipid peroxidation, iron(III) complexes with a salene scaffold were identified as potent ferroptosis inducers [24–26]. The full potential of these complexes, however, has not been reached yet, as they showed a relatively poor cellular uptake. In order to address this shortcoming, it was the aim of this study to design novel complexes of higher lipophilic character than those which had been tested so far [25]. Lipophilicity has long been recognized as a key determinant of cellular uptake since highly lipophilic complexes tend to permeate cell membranes more readily, granting them easier access to intracellular targets [27–30]. In addition, halogenated complexes may be beneficial in terms of cytotoxicity. Especially compounds containing fluorine are promising due to their hydrophobic and lipophobic behaviour [31]. By investigating structural modifications to increase lipophilicity, a better understanding of the correlation between lipophilicity, halogenation and cellular uptake of iron complexes shall be provided.

Therefore, novel halogenated chlorido[*N,N*′-bis(salicylidene)-1,2-bis(3-methoxyphenyl)ethylenediamine]iron(III) complexes with fluorine (**X1**), chlorine (**X2**) and bromine (**X3**) in 5- position in the salicylidene moieties were designed and carefully characterized. LogP values were calculated by two computational methods and determined by reversed-phase high-resolution liquid chromatography (HPLC). Cellular uptake was investigated by graphite-furnace atom absorption spectroscopy (GF-AAS). The stability of the complexes was analyzed with regard to reactivity toward nucleophiles. Moreover, the structure–activity relationship (SAR) of these complexes was investigated by proliferation and cytotoxicity assays on the MDA-MB 231 triple negative breast cancer and the non-cancerous SV-80 fibroblast, HS-5 stroma and MCF-10A mammary gland cell lines. Cell-death studies and live confocal microscopy detecting mitochondrial reactive oxygen species (mROS), a mode of action previously shown for iron(III) complexes [24–26, 32–35] were performed in order to discover the effect of these complexes on the cellular level.

## Materials and methods

### General

The chemical reagents and solvents were purchased from commercial suppliers (Sigma-Aldrich, Fluka, Alfa Aesar, Acros and VWR) and were used without further purification.

Analytical thin-layer chromatography: Polygram SIL G/UV254 (Macherey–Nagel) plates (0.25 mm layer thickness) with a fluorescent indicator and Merck TLC Silica gel 60 F 254 aluminium backed plates. The spots were visualized with UV light (254/365 nm).

Nuclear magnetic resonance (NMR) spectroscopy: Bruker Ultrashield 400 Plus spectrometer (^1^H NMR, 400 MHz; ^13^C NMR, 100 MHz). The centres of the solvent signal and the tetramethylsilane (TMS) signal were used as internal standards. Deuterated solvents used for the NMR spectra were purchased from Eurisotop (Saarbrücken, Germany). The chemical shifts are given in parts per million (ppm) and the coupling constants are given in Hertz (Hz).

Magnetic measurements of complexes **X1–X3** in solution were performed at room temperature (rt) by ^1^H NMR spectroscopy using the Evans method [36] on a Bruker Avance 400 spectrometer operating at 400.14 MHz at a constant temperature of 298.15 K. The measurements of each complex were performed in standard 5 mm NMR tubes containing the paramagnetic samples dissolved in deuterated dimethyl sulfoxide (DMSO-*d*_*6*_) with an inert reference of 0.03% TMS against a reference insert tube filled with the same solvent.

High-resolution mass spectrometry (HR-MS): An Orbitrap Elite mass spectrometer (Thermo Scientific, Waltham, MA, USA) using direct infusion and heated electrospray ionization (HESI) was employed. HR-MS data analysis was carried out with Xcalibur.

Elemental analysis (CHN): Measurements were performed at the Department of General, Inorganic and Theoretical Chemistry, University of Innsbruck, Austria with UNICUBE—Elementar, Langensbold, Germany.

Reversed-phase HPLC: Shimadzu Nexera-i-LC-2040C-3D using a RP-18 end capped 250–4 mm column.

Fourier transform infrared (FT-IR) spectroscopy: Bruker Alpha spectrometer with an ATR unit. FT-IR spectra were measured with 32 scans in the wavenumber range covering 4000 to 400 cm^−1^ and exerting a resolution of 1 cm^−1^.

Electron paramagnetic resonance (EPR) spectra were recorded on a Magnettech 5000 X-band spectrometer in a frozen solution of DMSO in 3 mm o.d. fused silica tubes at 98 K. Simulation was performed with the pepper function of the EasySpin package for Matlab [37].

GF-AAS: M6 Zeeman GFAA-Spectrometer (Thermo Scientific).

Live confocal microscopy: Zeiss Axio Observer Z1 instrument (Zeiss, Oberkochen, Germany).

### Chemistry

#### Synthesis of meso-1,2-bis(3-methoxyphenyl)ethylenediamine (II) (step 1)

6.193 mmol (1.513 g) of meso-1,2-bis(2-hydroxyphenyl)ethylenediamine **(I)** and 14.21 mmol (1.935 g) 3-methoxybenzaldehyde were stirred in acetonitrile anhydrous (MeCN) under reflux for 12 h to obtain after recrystallization a colourless powder. Yield: 5.129 mmol (1.397 g), 93%.

This intermediate was suspended in 50 ml MeCN and 12.5 ml 37% HCl (ratio 4:1) and stirred under reflux for 5 h. After cooling overnight, the formed precipitate was collected via filtration and washed with MeCN and dried in vacuo. Yield: 3.43 mmol (0.935 g), 88%, colourless powder.

#### General procedure for the synthesis of the ligands (step 2)

Briefly, a mixture of 1 equivalent (equiv.) of meso-1,2-bis(3-methoxyphenyl)ethylenediamine **(II)** and 2 equiv. of respectively substituted salicylaldehyde was dissolved in MeCN anhydrous (10 mL) and stirred under reflux for 48 h. After cooling overnight, the formed precipitate was collected via filtration and washed with ice-cold MeCN. After drying in vacuo, the ligands were obtained as a yellow powder. All ligands were characterized and the purity was verified based on HPLC confirming purity > 95% (Table S1). The spectra and the HPLC chromatograms are given in the supplementary information (Figures S1–S15).

#### [Meso-*N,N*′-bis(5-fluorosalicylidene)-1,2-bis(3-methoxyphenyl)ethylenediamine] (L1)

532 µmol (145 mg) meso-1,2-bis(3-methoxyphenyl)ethylenediamine **(II)** and 1.27 mmol (178 mg) of 5-fluoro-salicylaldehyde; Yield 99% (0.527 mmol, 272 mg); melting point (mp): 159 °C.

^1^H NMR (400 MHz, Chloroform-d) δ 12.81 (s, 2H, OH), 8.03 (s, 2H, HC=N), 7.22 (t, J = 7.9 Hz, 2H), 7.01 (ddd, J = 9.1, 8.1, 3.1 Hz, 2H), 6.92 to 6.84 (m, 4H), 6.84 to 6.75 (m, 6H), 4.70 (s, 2H, CH), 3.73 (s, 6H, OCH_3_).

^13^C NMR (101 MHz, Chloroform-d) δ 164.85 (d, J = 2.7 Hz, 2C), 159.70, 158.34 to 152.74 (m, 2C), 140.64 (2C), 129.68 (2C), 120.23 (2C), 119.75 (d, J = 23.3 Hz, 2C), 118.15 (dd, J = 36.9, 7.3 Hz, 4C), 116.74 (d, J = 23.2 Hz, 2C), 113.86 (2C), 113.30 (2C), 77.23 (2C), 55.22 (2C).

FT-IR: ν_max_ = 3600 w, 3069–2838 w, 1632 s, 1588 s, 1488 s, 1369 m, 1320 m, 1257 s, 1166 m, 1082 m, 1025 s, 959 m, 918 m, 870 m, 805 m, 784 s, 748 m, 699 s, 672 m, 613 m, 573 m, 461 m.

HR-MS: m/z (M + H)^+^: calculated: 517.1933; found: 517.1934.

CHN: calculated: C 69.76 H 5.07 N 5.42; found: C 69.73 H 5.21 N 5.54.

Purity calculated by HPLC (peak area): 96.4%

#### [Meso-*N,N*′-bis(5-chlorosalicylidene)-1,2-bis(3-methoxyphenyl)ethylenediamine] (L2)

558 µmol (152 mg) meso-1,2-bis(3-methoxyphenyl)ethylenediamine **(II)** and 1.28 mmol (200 mg) of 5-chloro-salicylaldehyde; Yield 76% (0.424 mmol, 233 mg); mp: 175 °C.

^1^H NMR (400 MHz, Chloroform-d) δ 13.04 (s, 2H, OH), 8.02 (s, 2H, HC=N), 7.26 to 7.19 (m, 4H), 7.05 (d, J = 2.5 Hz, 2H), 6.91 to 6.85 (m, 4H), 6.83 to 6.76 (m, 4H), 4.70 (s, 2H, CH), 3.74 (s, 6H, OCH_3_).

^13^C NMR (101 MHz, Chloroform-d) δ 164.76 (2C), 159.74 (2C), 159.41 (2C), 140.56 (2C), 132.51 (2C), 130.77 (2C), 129.74 (2C), 123.35 (2C), 120.19 (2C), 119.36 (2C), 118.50 (2C), 113.85 (2C), 113.33 (2C), 77.23 (2C), 55.23 (2C).

FT-IR: ν_max_ = 3600 w, 3063–2836 w, 1630 s, 1606 m, 1582 m, 1477 m, 1359 m, 1273 s, 1181 m, 1150 m, 1042 s, 911 m, 883 m, 863 m, 820 m, 780 s, 743 m, 694 s, 647 m, 460 m.

HR-MS: m/z (M + H)^+^: calculated: 549.1342; found: 549.1353.

CHN: calculated: C 65.58 H 4.77 N 5.10; found: C 65.54 H 4.89 N 5.06.

Purity calculated by HPLC (peak area): 95.7%

#### [Meso-*N,N*′-bis(5-bromosalicylidene)-1,2-bis(3-methoxyphenyl)ethylenediamine] (L3)

441 µmol (120 mg) meso-1,2-bis(3-methoxyphenyl)ethylenediamine **(II)** and 1.18 mmol (238 mg) of 5-bromo-salicylaldehyde; Yield 90% (0.395 mmol, 252 mg); mp: 188 °C.

^1^H NMR (400 MHz, Chloroform-d) δ 13.07 (s, 2H, OH), 8.01 (s, 2H, HC=N), 7.35 (dd, J = 8.8, 2.5 Hz, 2H), 7.22 (t, J = 7.8 Hz, 2H), 7.19 (d, J = 2.5 Hz, 2H), 6.88 (dd, J = 7.7, 1.3 Hz, 2H), 6.85 to 6.76 (m, 6H), 4.70 (s, 2H, CH), 3.74 (s, 6H, OCH_3_).

^13^C NMR (101 MHz, Chloroform-d) δ 164.67 (2C), 159.89 (2C), 159.75 (2C), 140.55 (2C), 135.31 (2C), 133.76 (2C), 129.76 (2C), 120.18 (2C), 119.99 (2C), 118.95 (2C), 113.84 (2C), 113.33 (2C), 110.20 (2C), 79.59 (2C), 55.23 (2C).

FT-IR: ν_max_ = 3600 w, 3062–2835 w, 1629 s, 1605 m, 1581 m, 1473 m, 1358 m, 1272 s, 1149 m, 1041 s, 882 m, 820 m, 742 m, 689 m, 550 m, 459 m.

HR-MS: m/z (M + H)^+^: calculated: 639.0312; found: 639.0327.

CHN: calculated: C 56.45 H 4.11 N 4.39; found: C 56.23 H 4.18 N 4.26.

Purity calculated by HPLC (peak area): 95.8%

#### General procedure of the synthesis of the iron(III) complexes (step 3)

Briefly, a mixture of 1 equiv. substituted meso-1,2-bis(3-methoxyphenyl)ethylenediamine ligands **L1–L3** and 1 equiv. iron(III) chloride anhydrous was dissolved in ethanol anhydrous (EtOH, 7 mL). Quickly the solution turned dark and the reaction was allowed to complete for 2 h under reflux conditions. The precipitate was recrystallized in EtOH and dried in vacuo. All synthesized complexes were characterized and the purity was verified based on HPLC, confirming purity > 99% (Table S2). The spectra and the HPLC chromatograms are given in the supplementary information (Figures S16–S28).

#### Chlorido[meso-*N,N′*-bis(5-fluorosalicylidene)-1,2-bis(3-methoxyphenyl)ethylenediamine]iron(III) (X1)

310 µmol (160 mg)** L1** and 490 µmol (80 mg) FeCl_3_; Yield 27% (83 µmol, 50 mg); mp: 232 °C.

FT-IR: ν_max_ = 2937–2835 w, 1601 s, 1545 s, 1491 m, 1461 s, 1376 m, 1281 s, 1220 m, 1148 m, 1045 m, 998 m, 875 m, 820 s, 779 m, 715 m, 697 m, 553 m, 524 m, 458 m, 432 m.

HR-MS: m/z (M–Cl)^+^: calculated: 570.1049; found: 570.1057.

CHN: calculated (X1 × EtOH): C 58.56 H 4.12 N 4.51; found: C 58.50 H 4.42 N 4.90.

μ_eff_ (Evans method, DMSO-*d*_6_) = 5.28 μ_B_.

EPR (9.5 GHz, 98 K) g_Ʇ_ = 4.16, g_ꟾꟾ_ = 7.96.

Purity calculated by HPLC (peak area): 99.9%

#### Chlorido[meso-*N,N′*-bis(5-chlorosalicylidene)-1,2-bis(3-methoxyphenyl)ethylenediamine]iron(III) (X2)

382 µmol (210 mg) **L2** and 520 µmol (85 mg) FeCl_3_; Yield 39% (150 µmol, 95 mg); mp: 253 °C.

FT-IR: ν_max_ = 3054–2835 w, 1611 s, 1532 m, 1488 m, 1457 s, 1381 m, 1303 m, 1259 s, 1177 m, 1154 m, 1038 m, 1001 m, 869 m, 843 m, 775 m, 716 m, 692 m, 665 m, 515 m, 486 s, 453 s.

HR-MS: m/z (M–Cl)^+^: calculated: 602.0457; found: 602.0447.

CHN: calculated: C 56.41 H 3.79 N 4.39; found: C 56.08 H 3.97 N 4.34.

μ_eff_ (Evans method, DMSO-*d*_6_) = 5.47 μ_B_.

EPR (9.5 GHz, 98 K) g_Ʇ_ = 4.14, g_ꟾꟾ_ = 7.91.

Purity calculated by HPLC (peak area): 99.8%

#### Chlorido[meso-*N,N′*-bis(5-bromosalicylidene)-1,2-bis(3-methoxyphenyl)ethylenediamine]iron(III) (X3)

287 µmol (183 mg) **L3** and 460 µmol (74 mg) FeCl_3_; Yield 48% (139 µmol, 101 mg); mp: 258 °C.

FT-IR: ν_max_ = 3054 to 2834 w, 1609 s, 1527 m, 1488 m, 1456 s, 1379 m, 1301 m, 1260 s, 1197 m, 1176 m, 1155 m, 1037 m, 1001 m, 842 m, 774 m, 712 m, 712 m, 652 s, 561 m, 512 m, 482 m, 446 m.

HR-MS: m/z (M–Cl)^+^: calculated: 691.9426; found: 691.9440.

CHN: calculated: C 49.52 H 3.32 N 3.85; found: C 49.18 H 3.34 N 3.77.

μ_eff_ (Evans method, DMSO-*d*_6_) = 5.39 μ_B_.

EPR (9.5 GHz, 98 K) g_Ʇ_ = 4.14, g_ꟾꟾ_ = 7.73.

Purity calculated by HPLC (peak area): 99.4%

#### Reversed-phase HPLC analysis

All runs were performed with a flow rate of 0.7 mL/min at 30 °C oven temperature. Sample preparation and experimental conditions: 1 mM solutions were prepared by dissolving the ligands in chloroform and the complexes in DMSO. Mobile phase: isocratic elution was performed with solvents purchased from VWR, HPLC grade with a mixture from 85% methanol and 15% H_2_O. Stationary phase: The Knauer Eurosher 100-5 reversed-phase C18 column with precolumn (250 × 4 mm, 5 µm) was used (Knauer, Berlin, Germany). A run was set to 30 min to ensure that any contamination could be detected. The first peak is corresponding to DMSO, the second peak is corresponding to the complexes. Detection and evaluation of the chromatograms was performed at 254 nm. All Figures were generated with OriginPro2018.

#### Reactivity toward biological nucleophiles

Aqueous stock solutions (10 mM in 18.2 MΩ·cm) of each amino acid (arginine (Arg), cysteine (Cys), histidine (His), lysine (Lys), methionine (Met)) as well as glutathione (GSH), were mixed with 1 equiv. of the respective stock solutions of complexes **X1–X3** (10 mM in DMSO) and diluted with H_2_O or 100 mM ammonium bicarbonate solution to a final concentration of 100 µM and left to incubate at rt. After 1 h, 24 h and 48 h an aliquot of the aqueous incubation mixture was diluted with H_2_O/MeCN containing 0.1% formic acid and investigated by HR-MS. The samples containing ammonium bicarbonate were analyzed after 24 h only. HR-MS spectra were recorded on an Orbitrap Elite mass spectrometer (Thermo Scientific) in positive ion mode. Typically, sample solutions were infused at 5 µL/min and ionized in the HESI source with standard conditions (HESI temperature 45 °C, 4 kV spray voltage, capillary temperature 275 °C and sheath gas flow rate at 5 arbitrary units). Data analysis was performed using the Xcalibur software package (Thermo Scientific).

### Biology

#### Cell lines and compounds

The mammary carcinoma cell line MDA-MB 231 was purchased from the German Collection of Microorganisms and Cell Cultures (DSMZ), Braunschweig, Germany. The non-cancerous cell line SV-80 was kindly provided by the Department of Hematology, Medical University of Innsbruck. The non-tumorous stroma cell line HS-5 was kindly provided by the Tyrolean Cancer Research Institute. The cell lines were grown in RPMI 1640 without phenol red (BioWhittaker, Lonza, Walkersville, MD, USA), supplemented with a solution of glutamine (2 mM), penicillin (100 U/mL), streptomycin (100 µg/mL; Sigma-Aldrich, St. Louis, MO, USA) and fetal bovine serum (FBS; 10%; Biowest, Nuaillé, France) at 37 °C in a 5% CO_2_ / 95% air atmosphere and fed twice weekly. The MCF-10A cell line was kindly provided by the Department of Gynecology, Medical University of Innsbruck. The cell line was cultivated in mammary epithelial cell growth medium bullet kit (Lonza) containing mammary epithelial cell basal medium supplemented with human epidermal growth factor (0.1%), bovine pituitary extract (0.4%), hydrocortisone (0.1%), insulin (0.1%), but without gentamycin sulfate. Furthermore, cholera toxin (100 ng/ml; Sigma-Aldrich) was added to the cell-culture medium. MCF-10A cells were also fed twice a week.

Ferrostatin-1 and Necrostatin-1 were purchased from Sigma-Aldrich, dissolved as a 10 mM stock solution in DMSO and stored at – 20 °C. All iron(III) salene complexes were also dissolved in DMSO as a stock solution of 10 mM and stored at rt. Cisplatin was dissolved in dimethylformamide (DMF) as a stock solution of 10 mM and stored at – 20 °C. On the day of complex addition, the stock solution was adjusted with the respective cell-culture medium to reach the test concentration. The concentration of the solvents did not exceed 0.1% and did not induce any activity.

#### Analysis of proliferation and metabolic activity

Logarithmically growing MDA-MB 231, SV-80, HS-5 and MCF-10A cells were resuspended in the respective cell-culture medium at 1 × 10^5^ cells/mL (MDA-MB 231, SV-80) or 0.7 × 10^5^ cells/mL (HS-5, MCF-10A), plated in triplicate in flat-bottomed 96-well plates (100 µL; Falcon, Corning Life Sciences, Durham, NC, USA) and incubated at 37 °C in a 5% CO_2_ / 95% air atmosphere for 24 h. Thereafter, complexes were added to reach the final concentrations in a total volume of 150 µL for further 72 h.

For the proliferation assay each well was exposed to 2 µCi [^3^H]-thymidine (Hartmann Analytic, Braunschweig, Germany) during the last 12–16 h of incubation. Cells were harvested using a semiautomated device, and the [^3^H]-thymidine uptake expressed in counts per minute (cpm) was measured in a scintillation counter (Microbeta Trilux, PerkinElmer, Waltham, USA).

Metabolic activity was analyzed using a modified 3-(4,5-dimethylthiazol-2-yl)-2,5-diphenyltetrazolium bromide (MTT) assay (EZ4U kit; Biomedica, Vienna, Austria) according to the manufacturer´s instructions. The proliferation and the metabolic activity in the absence of the complexes were set to 100% and the metabolic activity of the respective complex was calculated as percentage of the control (without complex).

#### Mitochondrial reactive oxygen species (mROS) staining

MDA-MB 231 were seeded at a density of 2 × 10^4^ cells per well on eight-well Nun Lab-Tek chambered coverglass dishes (Thermo Scientific) and incubated for 24 h. Thereafter, complexes were added and incubated for another 24 h. Shortly before analysis 5 µL of 20 µM HEPES buffer (Biochrom GmbH, Berlin, Germany) were added to each well. Subsequently, 2 µL of reduced MitoTrackerRed-H_2_XROS (Invitrogen; Thermo Scientific) were added and incubated for further 15 min. The cells were analyzed by confocal microscopy using an inverted microscope (Zeiss Axio Observer Z1, Zeiss, Oberkochen, Germany) in arrangement with a spinning disk confocal system (UltraVIEW VoX, PerkinElmer, Waltham, MA, USA).

#### Lipid peroxidation staining

Logarithmically growing MDA-MB 231 cells were resuspended in cell-culture medium at 1 × 10^5^ cells/mL and 100 µL thereof were seeded in triplicate in flat-bottomed 96-well plates. After a 24 h incubation at 37 °C in a 5% CO_2_ / 95% air atmosphere complexes were added at concentrations ranging from 0.25 µM to 2.5 µM. After a further incubation period of 2 h and 4 h cells were detached with accutase (Sigma-Aldrich), collected and centrifuged at 200 relative centrifugal force (rcf) for 5 min. The cell pellets were resuspended in a 2.5 µM BODIPY 581/591 staining solution (Invitrogen) and incubated for 30 min at 37 °C in the dark. After another centrifugation for 10 min at 200 rcf and 4 °C, each pellet was resuspended in 200 µL of PBS and immediately analyzed by flow cytometry on the FACSCanto II (Becton Dickinson, San Jose, CA, USA).

#### Cellular uptake

MDA-MB 231 cells (0.5 × 10^6^) were seeded in 25 cm^2^ flasks. After reaching 70–80% of confluence, the cell-culture medium was replaced by 3 mL of RPMI + 10% FBS containing the complexes at a final concentration of 2.5 μM. The flasks were incubated for 0 min, 1 h, 4 h, and 24 h, respectively. Thereafter, the cells were washed twice with 1 mL of phosphate buffered saline (PBS) and treated with accutase for 5 min. As soon as all cells detached from the bottom of the flask, 1 mL of cell-culture medium was added and the mixture was transferred to a 1.5 mL Eppendorf tube and centrifuged at 2300 rcf for 3 min at 4 °C. The cell pellets were washed twice with 1 mL of PBS and stored at – 20 °C until analysis. Directly after thawing the cell pellets were resuspended in Milli-Q water containing 0.2% Triton X-100 and lysed by sonication in a cup booster (Sonopuls, Bandelin, Berlin, Germany) three times for 120 s, with cooling at 4 °C cycle and 8.65% power.

The iron content of the cell pellets was determined by GF-AAS (M6 Zeeman GFAA-Spectrometer; Thermo Scientific) at 248.3 nm and Zeeman background correction using a 1100 °C ash temperature and a 2100 °C atomization temperature under an argon atmosphere.

The intracellular uptake is presented as the amount of pg Fe/µg protein referred to the cellular protein mass (μg) determined by a classical Bradford assay.

### Statistical analysis

The Mann Whitney U test was used to analyze the differences between proliferation, metabolic activity and lipid peroxidation in the absence and the presence of a variable concentration of the test complexes and the values of the compound treated non-tumorous cell lines against the mammary carcinoma cell line (NCSS software, Kaysville, UT, USA).

IC_50_ values were calculated with Quest Graph IC_50_ Calculator from AAT Bioquest, Inc.

## Results and discussion

### Chemistry

#### Synthesis of the complexes

**Scheme 1:**
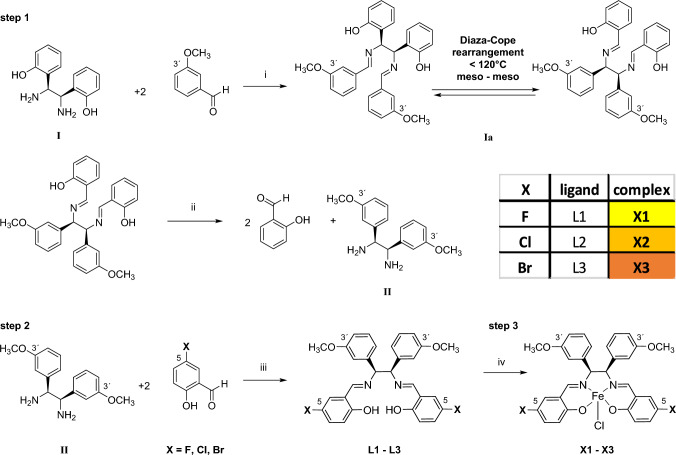
Synthetic pathway for the formation of chlorido[*N,N*′-bis(salicylidene)-1,2-bis(3-methoxyphenyl)ethylenediamine]iron(III) complexes with fluorine, chlorine and bromine substituents in the position 5 in the salicylidene moieties. Step 1: synthesis of meso-1,2-bis(3-methoxyphenyl)ethylenediamine (II): Reagents and conditions: (i) MeCN anhydrous, reflux 7 h, Diaza-Cope rearrangement of **(Ia)** [38]; (ii) acid hydrolysis MeCN:HCl 37% 4:1, rt, 17 h, 6 M NaOH. Step 2: Synthesis of the ligands **L1**–**L3** with fluorine, chlorine and bromine substituents in position 5, respectively. (iii) MeCN anhydrous, reflux, 1–5 h. Step 3: Synthesis of iron(III) complexes **X1**–**X3**: (iv) FeCl_3_ ethanol anhydrous, reflux, 0.5–2 h

The synthesis of halogenated chlorido[*N,N*′-bis(salicylidene)-1,2-bis(3-methoxyphenyl)ethylenediamine]iron(III) complexes **X1–X3** was performed in three steps, as described by our research group previously [25], and is depicted in Scheme 1.

In the first step, the meso-1,2-bis(3-methoxyphenyl)ethylenediamine moiety **(II)** was prepared. Initially, one equiv. of meso-1,2-bis(2-hydroxyphenyl)ethylenediamine **(I)** and two equiv. of 3-methoxybenzaldehyde were combined and stirred in anhydrous MeCN, forming a Schiff base intermediate **(Ia)**.

Next, the intermediate **(Ia)** underwent a stereoselective meso–meso Diaza-Cope rearrangement reaction [38]. The resulting diimine was cleaved by acid hydrolysis in a mixture of MeCN:HCl (ratio 4:1) to obtain meso-1,2-bis(3-methoxyphenyl)ethylenediamine **(II)**. In the second step, this product was reacted with 5-fluoro-, 5-chloro- and 5-bromo-salicylaldehyde, respectively, yielding yellow-coloured ligands **L1–L3** in high yield.

These ligands were characterized by ^1^H, ^13^C NMR and HSQC spectroscopy, HR-MS and FT-IR spectroscopy (Figures S1–S12). The elemental composition was confirmed by CHN and purity by HPLC (Table S1, Figures S13–S15).

In the third step, complexation with iron(III) chloride was performed. The strong coordinative nature of the iron ion facilitated rapid complexation, resulting in a noticeable colour change from yellow to black in solution. All complexes (**X1–X3**) were isolated as black powder and analyzed with FT-IR (Figures S16–S18) and HR-MS. The elemental composition of the complexes was also corroborated by CHN. In addition, the purity of all complexes was confirmed by HPLC showing a retention time between 4.3 and 9.6 min with a purity of at least 99.4% (Table S2, Figures S19–S21). The paramagnetism of the iron(III) salene complexes was evaluated by Evans method [36], revealing a magnetic moment of 5.28 μ_B_, 5.47 μ_B_ and 5.39 μ_B_, for **X1, X2** and **X3**, respectively (Figures S22–S27). This is in line with other reported iron(III) complexes [25, 39, 40]. Obtained values were close to the spin-only value of 5.92 μ_B_ for S = 5/2, indicating the formation of high-spin iron(III) complexes. Furthermore, electron paramagnetic resonance (EPR) was simulated and measurements of all complexes agree with metal centered S = 5/2 spin systems and detected the expected g-values for **X1–X3** (Figure S28).

#### Reactivity toward biological nucleophiles

**Fig. 1:**
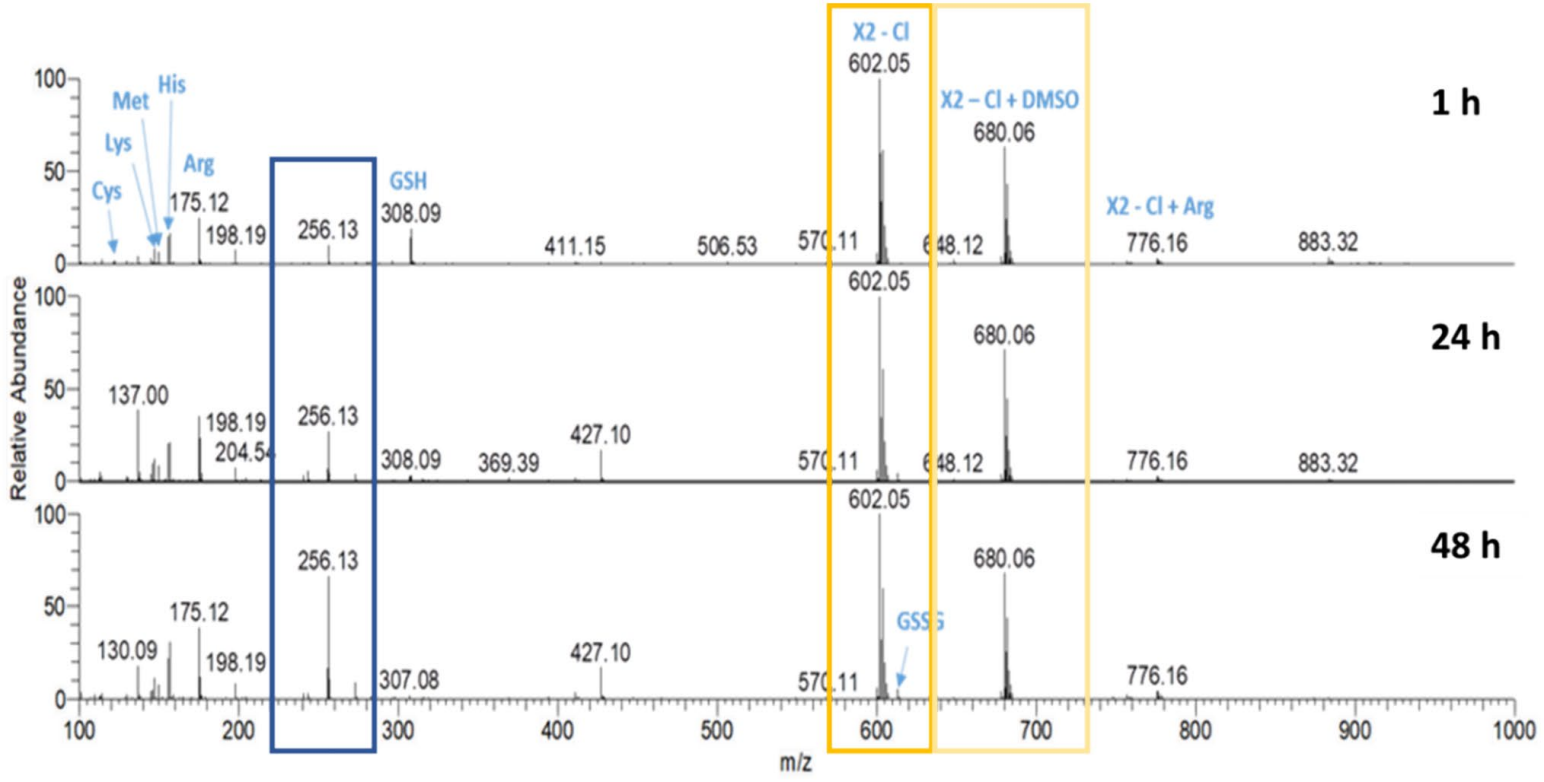
HR-MS spectra of **X2** in an aqueous solution containing equimolar amounts of Arg, Cys, His, Lys, Met and GSH analyzed for 1 h, 24 h and 48 h

**Fig. 2:**
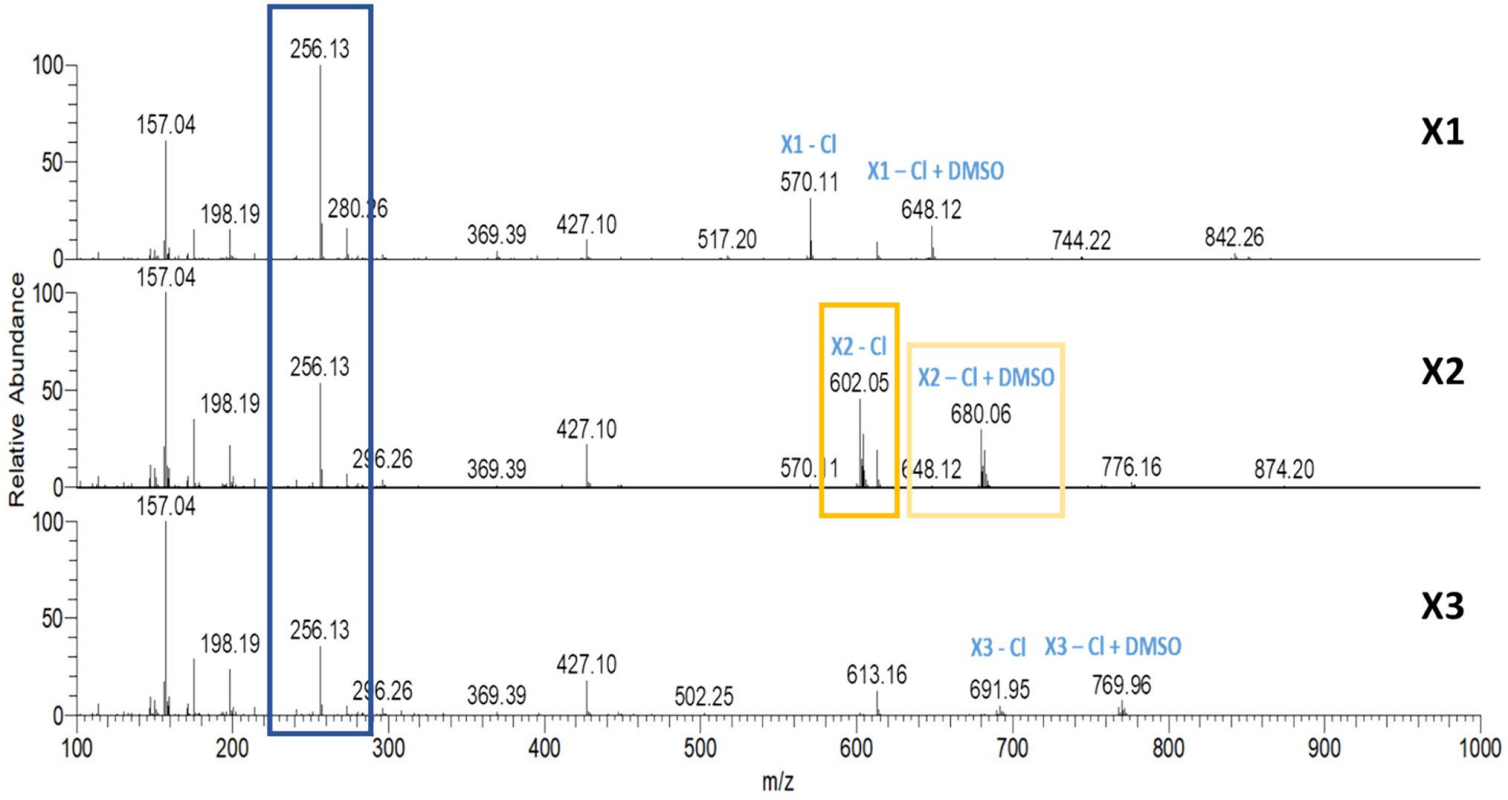
HR-MS spectra of **X1–X3** in a 100 mM ammonium bicarbonate solution containing equimolar amounts of Arg, Cys, His, Lys, Met and GSH analyzed after 24 h

Many metal-based drugs are known to interact with biomolecules through formation of coordinate bonds. Nucleophilic sites in deoxyribonucleic acid (DNA) or amino acid side chains like amines, thioethers, thiols and carboxylates have been shown to form adducts with metal complexes [27, 41–44]. Cisplatin, a widely employed chemotherapeutic agent and a prominent metal complex, exerts its mode of action through dative bond formation with guanine bases in DNA. Many of its severe side effects, however, have been attributed to additional interactions with enzymes and other biomolecules [45, 46]. Therefore, a HR-MS study was performed to understand the reactivity and stability of **X1, X2** and **X3** in the presence of biologically relevant nucleophiles, which include the amino acids Arg, Cys, His, Lys, Met and GSH. These biological nucleophiles were each incubated in a 1:1 ratio with the complexes **X1–X3** (stock solution in DMSO 10 mM) in Milli-Q water (pH 6.0) or 100 mM ammonium bicarbonate solution (pH 7.5). The reactivity toward the provided biological nucleophiles was monitored in aqueous solution for 1 h, 24 h and 48 h and in ammonium bicarbonate solution for 24 h. The HR-MS spectra of **X2** in aqueous solution at various time points are shown in Fig. 1 and Table 1 lists the ions detected. The HR-MS spectra of **X1** and **X3** are presented in Figures S29–S30 and Table S3. As reported previously [26], the chlorido ligand was partially exchanged for DMSO (m/z 680, framed in ocher) which was used as solvent to prepare the stock solutions, or lost in the HESI process (m/z 602, framed in yellow). Ions representing the intact **X1–X3** complexes remained unchanged high abundant signals for 48 h. Negligible adduct formation was detected with the tested biomolecules in both Milli-Q water (Figs. 1, S29, S30) and ammonium bicarbonate solution (Fig. 2). These data indicate that, unlike many other metal-based drugs, complexes **X1–X3** exhibit pronounced stability against biological nucleophiles.

**Table 1:**
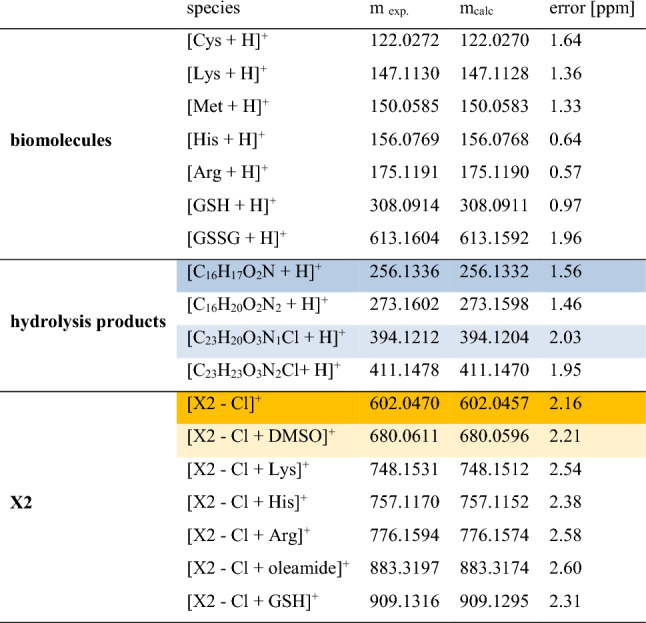
List of ions detected by HR-MS analysis of **X2** in aqueous solution

Oleamide is a common contaminant

**Fig. 3:**
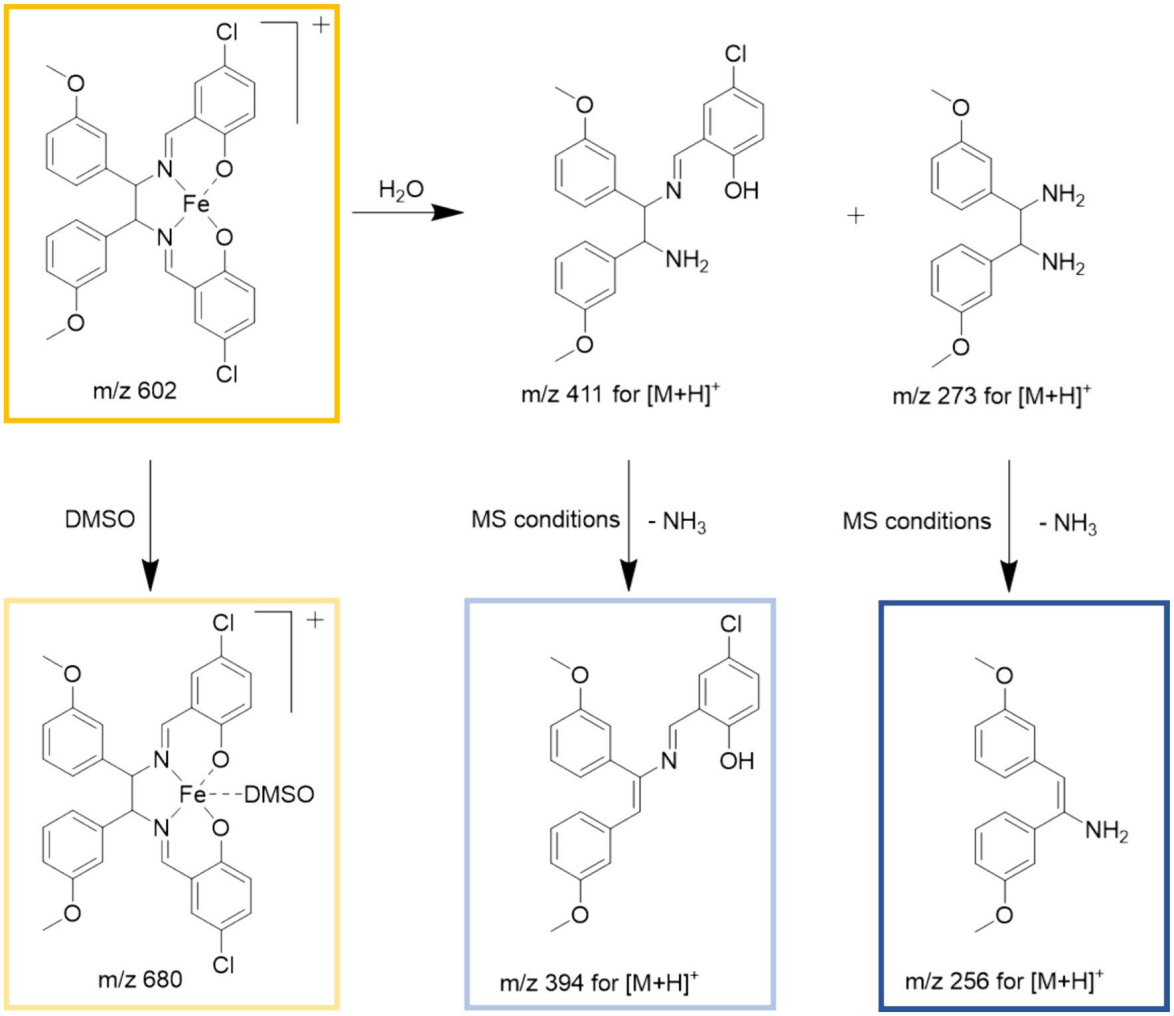
Ligand exchange and partial hydrolysis leads to a number of signals in the HR-MS spectra

Complexes **X1**–**X3**, after loss of the chlorido ligand, labeled **X1** – Cl, **X2** – Cl, **X3** – Cl, respectively, and their DMSO adducts, labeled **X1** – Cl + DMSO, **X2** – Cl + DMSO and **X3** – Cl + DMSO, respectively, remained the main species in HR-MS spectra over 48 h (Figs. 1, S29, S30 and Table S3). Only some very low abundant signals of adducts with amino acids were detected (Figs. 1 and 2, Tables 1, S3). Nonetheless, there was no observable interaction with amino acids containing thiol groups. Furthermore, certain hydrolysis products such as m/z 256 (framed in blue in Figs. 1, 2, 3, marked in blue in Table 1) and m/z 394 (marked in light blue in Table 1) were identified in both incubation solutions (Tables 1, S3). Nevertheless, it appeared that hydrolysis occurred at a swifter rate in the bicarbonate solution. Figure 3 provides a comprehensive representation of the signals observed in the HR-MS spectra.

#### Calculation of clogP values

To determine the increase in lipophilicity of the iron(III) complexes by the introduction of halogens into the salicylidene moieties two computational methods (Chemdraw 22.0.0 and DataWorrier Version 5.5.0) were applied. Although the values from DataWarrior and Chemdraw differ, because of their underlying algorithms, results of clogP derminations show the same trend (Table 2).

**Table 2:** clogP values of complexes **X1–X3** calculated with Chemdraw and DataWarrier

Complexes	Functional group	Chemdraw clogP	DataWarrier clogP
X1	F	7.1	5.1
X2	Cl	8.2	6.1
X3	Br	8.5	6.3

Indeed, the change of fluorine to chlorine increases the lipophilicity. After change of chlorine for bromine the highest values were calculated. The higher clogP values indicate a greater potential for partitioning into lipid-rich environments.

Moreover, the logP rank order of these complexes was evaluated experimentally. In reversed-phase HPLC, there is a direct correlation between logP and retention time: typically, compounds with higher logP values show longer retention times. The stationary phase is usually hydrophobic and the mobile phase comparatively more polar, compounds with higher logP values (indicating greater hydrophobicity) tend to exhibit longer retention times due to their enhanced interactions with the hydrophobic stationary phase. Conversely, compounds with lower logP values (indicating greater hydrophilicity) demonstrate shorter retention times because they have less interaction with the stationary phase, resulting in faster elution. Thus, the assessed retention times for complexes **X1**–**X3** can be correlated with their respective logP values, establishing the rank order for the complexes.

Specifically, **X1** (with a retention time of 4.291 min) has the lowest logP, followed by **X2** (7.614 min), and **X3** (9.634 min). This pattern correlates with their chemical structures and calculated clogP values, where **X1**, containing a fluorine atom, exhibits the lowest clogP, followed by **X2** with a chlorine atom, and **X3** with a bromine atom, indicating the highest clogP in this series.

In addition to an increased lipophilic character provided by halogenation, the lipophobic properties of these functional groups per se will, especially fluorine, contribute to the improved membrane permeability of these iron(III) salene complexes. Since halogens are both hydrophobic and lipophobic exhibiting phase separation in both water and lipophilic solvents, they limit also lipophilic interactions of these complexes with phospholipids in the cell membrane during the permeation process [47].

#### Cellular uptake

To investigate if higher lipophilicity results also in an enhanced complex uptake the cellular iron content was determined by GF-AAS.

**Fig. 4:**
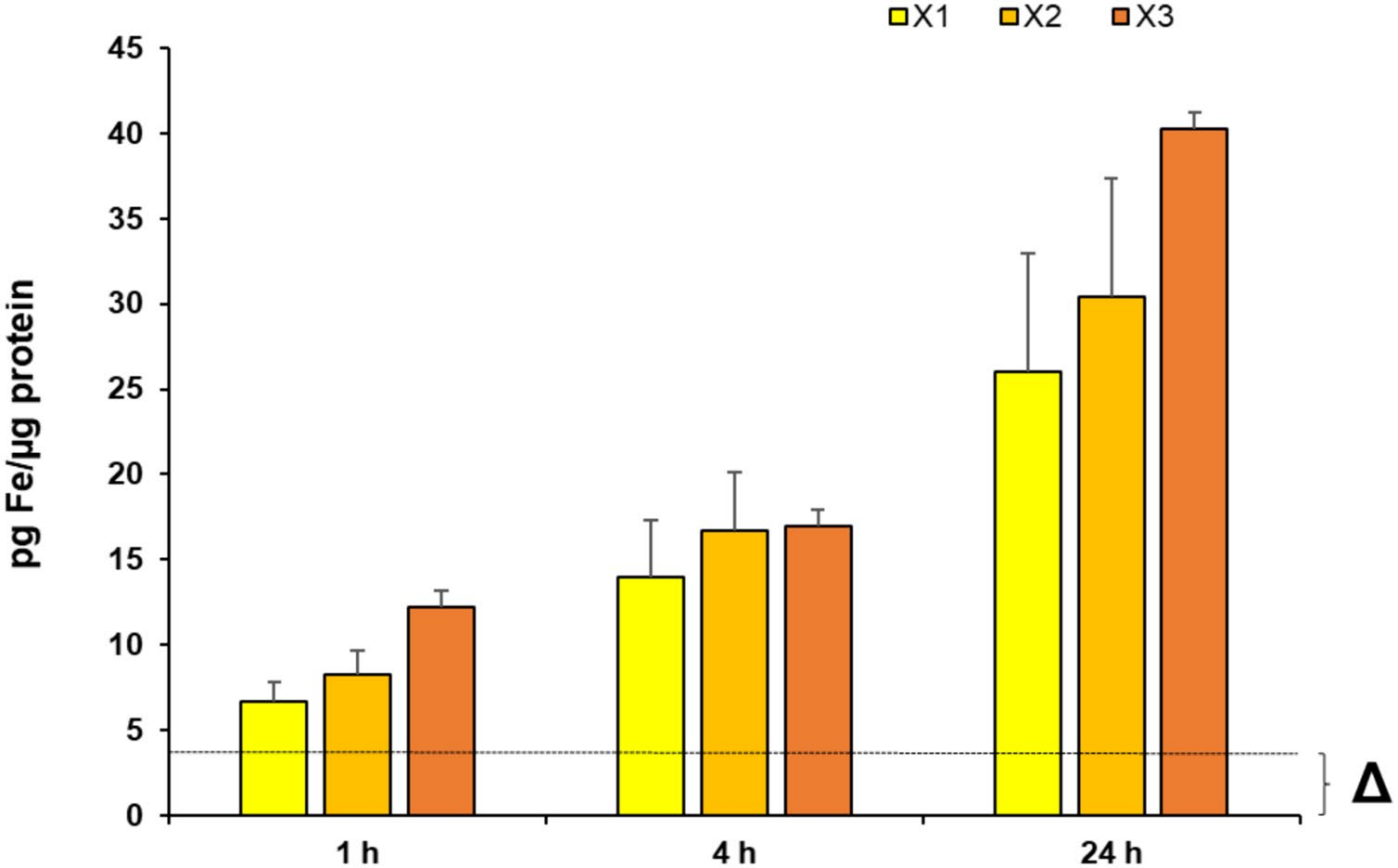
Cellular uptake measured via GF-AAS after 1 h, 4 h, and 24 h incubation of MDA-MB 231 cells with complexes **X1**–**X3** at 2.5 µM, respectively. MDA-MB 231 cells without addition of a complex (**Δ**) served as reference. Data are expressed as mean + SD of two independent experiments, measured in triplets

MDA-MB 231 cells were incubated with complexes **X1–X3** at a concentration of 2.5 µM for 1 h, 4 h and 24 h, respectively. To compensate the constitutive cellular iron content, cells without addition of a complex were prepared under identical conditions and served as negative control (**Δ**). The fluorine containing complex **X1** exhibited the comparatively lowest accumulation in the breast cancer cell line, followed by the chlorine-containing complex **X2** (Fig. 4).

The bromine-containing complex **X3** demonstrated the highest cellular uptake. The cellular uptake increased from 12.2 ± 2.5 pg Fe/µg protein (1 h) to 16.9 ± 3.7 pg Fe/µg protein (4 h) and finally to 40.2 ± 3.8 pg Fe/µg protein (24 h). These findings correlate perfectly with the logP rank order revealing the bromine complex (**X3**) as the most lipophilic complex, followed by chlorine (**X2**) and fluorine (**X1**).

### Biological activity

To evaluate whether enhanced lipophilicity and cellular uptake result in greater biological activity, we investigated the impact of the complexes on key parameters of antitumor activity, such as inhibition of proliferation and metabolic activity, in MDA-MB 231 cells. The focus on these well-established methods aimed to provide a more comprehensive understanding of how the complexes affect cell behaviour.

Accordingly, MDA-MB 231 cells were treated with varying concentrations of the complexes, ranging from 0.25 to 2.5 µM, and observed over a 72 h incubation period. Cisplatin served as a reference compound at a standard concentration of 1 µM in the proliferation and the metabolic activity analysis. This concentration was selected based on prior studies conducted within our research group, which investigated the specific effects of Cisplatin on MDA-MB 231 cells [25, 26].

#### Inhibition of proliferation

**Fig. 5:**
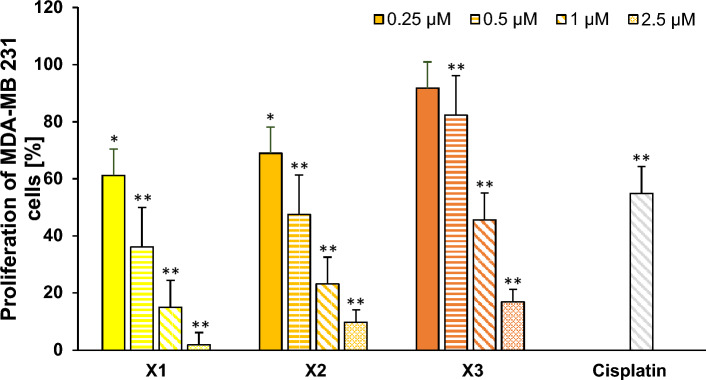
Effect of the iron(III) salene complexes **X1–X3** on the proliferation of MDA-MB 231 cells. Cisplatin at 1 µM was used as a reference. The mean proliferation + SE of eight independent experiments is plotted. The proliferation of cells only (without complex) was set at 100% (data not shown). The asterisks (*p < 0.01 and **p < 0.0005 against no complex) represent statistical significance

The influence on cell proliferation was determined by measuring [^3^H]-thymidine uptake into the DNA of dividing cells. All complexes demonstrated a significant, concentration dependent decrease of the proliferation of MDA-MB 231 cells in the low µM range (Fig. 5).

At higher concentrations **X1** inhibited the proliferation stronger than **X2** (reductions at 1 µM and 2.5 µM: **X1** to 15.1 ± 4.3% and 1.9 ± 0.4%, respectively, and to 23.3 ± 9.9% and 9.8 ± 6.4%, respectively, for **X2**). **X3** showed approximately two times lower effects, reducing proliferation to 16.9 ± 4.5% at 2.5 µM. Comparison of the proliferation inhibition at 1 µM revealed that **X1** outperformed Cisplatin 3.6-fold, **X2** 2.3-fold and **X3** 1.2-fold (Fig. 5).

#### Inhibition of metabolic activity

As proliferation inhibition is mostly accompanied by cytotoxicity, the metabolic activity of MDA-MB 231 cells was determined photometrically by evaluating the function of living mitochondria which correlates with cytotoxicity. This was achieved by measuring the reduction of light-yellow tetrazolium salt into orange formazan derivative after a 72 h incubation period.

Similar to the results from the proliferation assay, the complexes induced a concentration dependent inhibition of metabolic activity of MDA-MB 231 cells (Figure S31). **X1** demonstrated the highest efficacy in reducing metabolic activity. At 0.25 µM and 2.5 µM, **X1** reduced metabolic activity to 59.8 ± 9.5% and 16.3 ± 8.3%, respectively.

The cytotoxic activity of **X2** and **X3** at 2.5 µM was comparable, with inhibition of metabolic activity to 14.5 ± 7.4% and 16.2 ± 4.7%, respectively. In agreement with the results of cell proliferation, 1 µM of the complexes also outperformed Cisplatin in this analysis 3.8-fold in the case of **X1**, 1.9-fold in the case of **X2** and 1.2-fold in the case of **X3**.

**Scheme 2:**
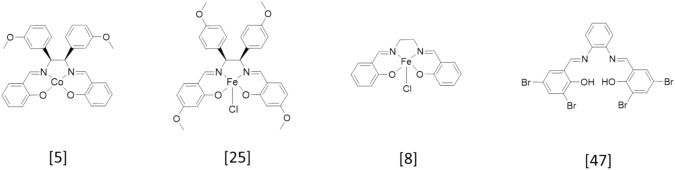
Overview of anticancer compounds used for comparison of IC_50_ values

Due to the lack of structural similarities, directly comparing the antitumor activity of **X1**–**X3** with other compounds (Scheme 2) is challenging. Substituting iron for cobalt to yield compound m8 [5], resulted in a 45% inhibition of the mammary carcinoma cell line MCF-7 (at 10 µM), which is clearly lower than the inhibitory capacity of **X1**–**X3**. Comparing the compounds of this series with C2, which displays a methoxy group in position 4 in the salicylidene moieties and shows an IC_50_ of 4.2 µM at MDA-MB 231 cells [25], a clear advantage of introducing halogens can be deduced. Concerning the iron(III) salene scaffold, Ansari et al. [8] reported on compound 1, which had an IC_50_ of 22 µM for MCF-7 cells and 31 µM for MCF-10A cells. The effect of bromine substitution in salophene complexes on antitumor activity was investigated by Zelada-Guillén et al. [48] on various cancer cell lines. Even the best compound (3) displayed an IC_50_ of 9.6 µM on PC-3 prostatic adenocarcinoma cells, which is clearly higher than the IC_50_ of **X1**–**X3**. Taken together, these comparisons underscore the improvement of antitumor activity by compounds with higher lipophilicity.

The selective targeting of cancer cells is critical for developing effective cancer therapies with minimal side effects. To evaluate the specificity of the new complexes, their cytotoxic effects were assessed on the non-cancerous fibroblast cell line SV-80, the non-tumorous stromal cell line HS-5, and the non-tumorous MCF-10A mammary gland cell line. These assessments, conducted after 72 h of incubation, offer a more accurate knowledge on the selectivity of the complexes toward cancer cells.

**Fig. 6:**
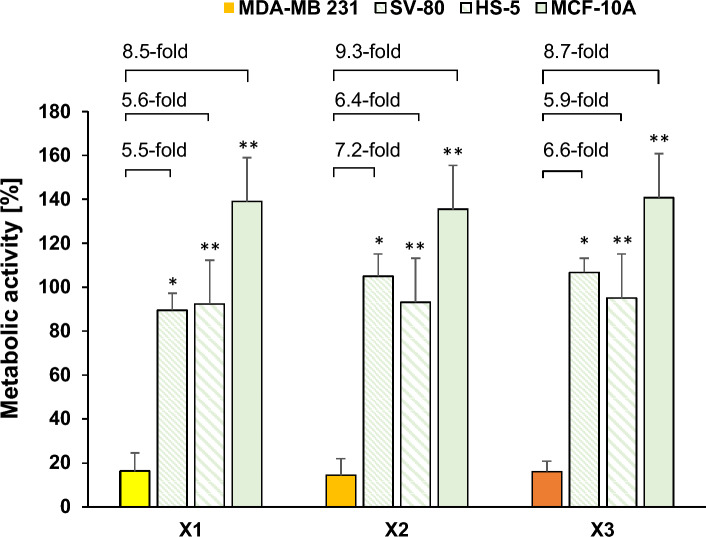
Metabolic activity of MDA-MB 231 cells incubated with **X1**–**X3** (yellow to orange) versus non-cancerous SV-80 (green, diagonally downwards), HS-5 (green, diagonally far down) and MCF-10A cells (green, bold). Values were calculated as a mean of metabolic activity + SE after a 72 h exposure with **X1**–**X3** at 2.5 µM (n = 4). The asterisks (*p < 0.05 and **p < 0.005 against the respective complex at 2.5 µM) represent statistical significance

Remarkably, none of the tested complexes displayed cytotoxicity against these non-cancerous cells, even at the highest concentration tested (2.5 µM; Fig. 6, Figures S32-S34). Among the complexes, **X2** demonstrated the greatest specificity, exhibiting a 7.2-fold higher cytotoxicity in the MDA-MB 231 breast cancer cells compared to the SV-80 fibroblast cells. This was followed by complex **X3** with a 6.6-fold difference, and complex **X1** with a 5.5-fold difference.

The same order of selectivity was observed in the other non-tumorous cell lines. For HS-5 cells, the differences were 6.4-fold, 5.9-fold, and 5.6-fold for complexes **X2**, **X3**, and **X1**, respectively. Similarly, in MCF-10A cells, the fold changes were 9.3, 8.7, and 8.5 for **X2**, **X3**, and **X1**, respectively. These findings indicate that the complexes have a high degree of specificity for the MDA-MB 231 breast cancer cell line, suggesting their potential utility in targeting cancer cells while sparing non-cancerous cells.

#### Production of mROS

The induction of mROS is one of the modes of action of iron(III) complexes [8, 24–26]. Therefore, MDA-MB 231 cells were incubated with **X1 – X3** for 24 h and thereafter stained with reduced MitoTrackerRed-H_2_XROS to detect mROS.

**Fig. 7:**
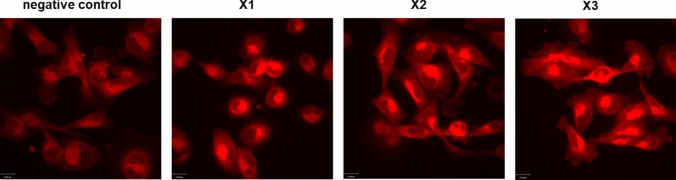
Induction of mROS in MDA-MB 231 cells without complex (negative control) and after treatment with **X1–X3** (0.5 μM) for 24 h and staining with reduced MitoTrackerRed-H_2_XROS (red)

Despite the limitations of live confocal microscopy in providing quantitative data, the images unambiguously revealed an increase in mROS production in cells treated with 0.5 µM of **X1–X3**, respectively, as compared to control cells (Fig. 7).

#### Induction of lipid peroxidation

Due to their high amount of polyunsaturated fatty acids, cellular membranes are very sensitive to excess ROS production, a process called lipid peroxidation. Indeed, iron(III) complexes have been shown to induce this mechanism of action [25, 26]. Therefore, lipid peroxidation was determined in MDA-MB 231 cells with the complexes at various concentrations by BODIPY 581/591 staining and subsequent analysis by flow cytometry. The incubation time was set to 2 h and 4 h, as these time points showed the highest lipid peroxidation in a previous study [25]. **X1–X3** induced lipid peroxidation to a limited extent, although statistically significant (Table 3). In general, the highest lipid peroxidation was induced with the highest concentration (2.5 µM) of the complexes.

**Table 3:** Percentage of cells displaying lipid peroxidation after a 2 h and 4 h incubation of MDA-MB 231 cells with **X1–X3** at concentrations ranging from 0.25 µM to 2.5 µM

Compound	Concentration (µM)	Percentage of cells with lipid peroxidation	
		2 h	4 h
Without compound		0.1 ± 0.0	0.1 ± 0.0
**X1**	0.25	0.4 ± 0.1	0.7 ± 0.3*
	0.5	0.7 ± 0.3*	0.9 ± 0.3*
	1	0.8 ± 0.2*	0.8 ± 0.3*
	2.5	1.2 ± 0.2*	1.1 ± 0.2*
**X2**	0.25	0.5 ± 0.3	0.7 ± 0.2*
	0.5	0.4 ± 0.1*	0.7 ± 0.2*
	1	0.4 ± 0.1*	1.0 ± 0.3*
	2.5	0.6 ± 0.1*	0.6 ± 0.2*
**X3**	0.25	0.5 ± 0.2*	0.5 ± 0.2*
	0.5	0.2 ± 0.0*	0.6 ± 0.2*
	1	0.4 ± 0.2*	0.6 ± 0.2*
	2.5	0.7 ± 0.1*	0.5 ± 0.3*

The mean percentage of cells ± SE with lipid peroxidation of five experiments is shown. The asterisk (*p < 0.05 against the cells without compound addition) represents statistical significance

#### Analysis of cell death

Considering the mitochondrial role in cell-death regulation [49] and slightly enhanced lipid peroxidation, an inhibitor study was performed to discriminate between the two main types of cell death, ferroptosis and necroptosis, induced by iron(III) complexes, as described before [24–26].

We added the ferroptosis inhibitor Ferrostatin-1 (Fer-1, 1 µM) and/or the necroptosis inhibitor Necrostatin-1 (Nec-1, 20 µM) concomitantly with **X1**, **X2** and **X3**, respectively, to MDA-MB 231 cells and analyzed their capacity to prevent the antimetabolic activity of the complexes.

**Fig. 8:**
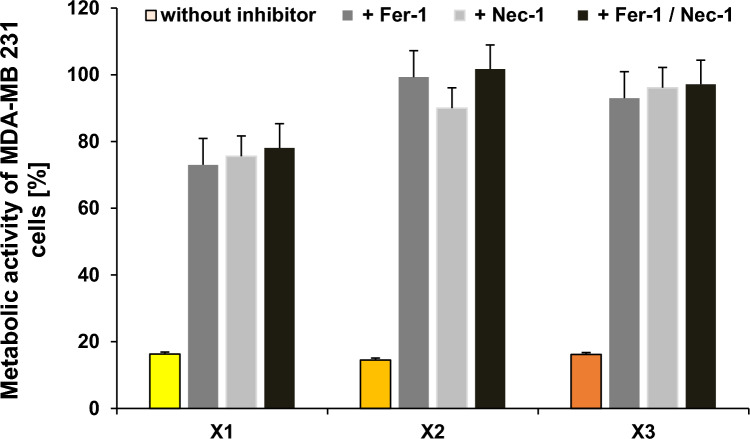
Effect of **X1–X3** (2.5 µM) in the absence and the presence of ferroptosis and/or necroptosis inhibitors Ferrostatin (Fer-1) and Necrostatin (Nec-1; dark and light grey or black) in MDA-MB 231 cells. The mean metabolic activity + SE of four independent experiments measured after 72 h of incubation is plotted

In agreement with the high mROS production and the low lipid peroxidation, both Fer-1 and Nec-1 effectively antagonized the inhibitory effects of all tested complexes, indicating the involvement of both ferroptosis and necroptosis in the cytotoxic effects of this series of iron(III) salene complexes (Fig. 8).

## Conclusion

To summarize, we successfully synthesized and investigated novel chlorido[meso-*N*,*N*′-bis(salicylidene)-1,2-bis(3-methoxyphenyl)ethylenediamine]iron(III) complexes with fluorine, chlorine and bromine substituents and correlated the lipophilicity with cellular uptake.

We evaluated the impact of these complexes on the MDA-MB 231 breast cancer cell line focusing on well-established cellular assays, proliferation and metabolic activity. Notably, complexes **X1–X3** at 1 µM outperformed Cisplatin, exhibiting up to 3.6-fold higher efficacy in proliferation inhibition and up to 3.8-fold higher effectiveness in metabolic activity reduction. The strong cytotoxic activity at 2.5 µM is attributed to high cellular uptake, which in turn corresponds to the increased lipophilicity. Through inhibition studies, we uncovered a dual mechanism of cell death induced by these complexes, involving both ferroptosis and necroptosis. Particularly remarkable was the selective targeting of the mammary carcinoma cell line while sparing non-malignant fibroblast, stroma and mammary gland cell lines.

Stability of the iron(III) salene complexes was proven by their lacking interaction with relevant biologically nucleophiles such as amino acids and GSH.

In light of these findings, the chlorido[meso-*N,N*′-bis(salicylidene)-1,2-bis(3-methoxyphenyl)ethylenediamine]iron(III) complexes with fluorine, chlorine and bromine emerge as promising lead structures for further optimization as anticancer drug candidates.

## Supplementary Information

Below is the link to the electronic supplementary material.

Supporting Information: 1H, 13C NMR, HSQC, FT-IR spectra and HPLC chromatograms of L1 − L3; FT-IR spectra, HPLC chromatograms, Evans 1H-NMR and EPR simulation and EPR spectra of X1 – X3, reactivity toward biological nucleophiles, biological activity. Supplementary file1 (PDF 1899 KB)

## Data Availability

The authors declare that the data supporting the findings of this study are available within the paper and its Supplementary Information files. Should any raw data files be needed in another format they are available from the corresponding author upon reasonable request.

## Abbreviations

cpm: Counts per minute
DMF: Dimethylformamide
DMSO: Dimethyl sulfoxide
DNA: Deoxyribonucleic acid
equiv.: Equivalent
EPR: Electron paramagnetic resonance
EtOH: Ethanol
FBS: Fetal bovine serum
Fer-1: Ferrostatin-1
FT-IR: Fourier transform infrared
GF-AAS: Graphite furnace atomic absorption spectrometry
GPx4: Glutathione peroxidase 4
HESI: Heated electrospray ionization
HPLC: High performance liquid chromatography
HR-MS: High-resolution mass spectrometry
HSQC: Heteronuclear single quantum correlation
Hertz: Hz
MeCN: Acetonitrile
m: Mitochondrial
min: Minute
mp: Melting point
MTT: 3-(4:5-Dimethylthiazol-2-yl)-2:5-diphenyltetrazolium bromide
Nec-1: Necrostatin-1
NMR: Nuclear magnetic resonance
PBS: Phosphate buffered saline
ppm: Parts per million
rcf: Relative centrifugal force
ROS: Reactive oxygen species
RPMI: Rosewell Park Memorial Institute medium
rt: Room temperature
SAR: Structure–activity relationship
SD: Standard deviation
SE: Standard error
TMS: Tetramethylsilane
