# Supplementary material for: Design, synthesis, and biological evaluation of novel halogenated chlorido[N,N′-bis(salicylidene)-1,2-bis(3-methoxyphenyl)ethylenediamine]iron(III) complexes as anticancer agents
Source: J Biol Inorg Chem. 2024 Aug 12;29(6):583–99. doi: 10.1007/s00775-024-02067-9 (PMC11390779; doi:10.1007/s00775-024-02067-9)
Supplement: Supplementary file 1 — Supporting Information: 1H, 13C NMR, HSQC, FT-IR spectra and HPLC chromatograms of L1 − L3; FT-IR spectra, HPLC chromatograms, Evans 1H-NMR and EPR simulation and EPR spectra of X1 – X3, reactivity toward biological nucleophiles, biological activity. Supplementary file1 (PDF 1899 KB) [file 775_2024_2067_MOESM1_ESM.pdf]

Supplementary Information

Design, synthesis, and biological evaluation of novel halogenated chlorido[*N*,*N′*-bis(salicylidene)-1,2-bis(3-methoxyphenyl)ethylenediamine]iron(III) complexes as anticancer agents

Astrid Dagmar Bernkop-Schnürch[1], Klaus Huber[1], Armida Clauser[1,2], Monika Cziferszky[1], Daniel Leitner[3], Heribert Talasz[4], Martin Hermann[5], Stephan Hohloch[3], Ronald Gust[1], Brigitte Kircher[*2,6]

[1]Department of Pharmaceutical Chemistry, Institute of Pharmacy, CMBI—Center for Molecular Biosciences Innsbruck, CCB—Center for Chemistry and Biomedicine, University of Innsbruck, Innrain 80-82, 6020 Innsbruck, Austria

[2]Immunobiology and Stem Cell Laboratory, Department of Internal Medicine V (Hematology and Oncology), Medical University of Innsbruck, Anichstraße 35, 6020 Innsbruck, Austria

[3]Department of General, Inorganic and Theoretical Chemistry, University of Innsbruck, Innrain 80-82, 6020 Innsbruck, Austria

[4]Biocenter, Institute of Medical Biochemistry, Protein Core Facility, Medical University of Innsbruck, Innrain 80-82, 6020 Innsbruck, Austria

[5]Department of Anesthesiology and Critical Care Medicine, Medical University of Innsbruck, Anichstraße 35, 6020 Innsbruck, Austria

[6]Tyrolean Cancer Research Institute, Innrain 66, 6020 Innsbruck, Austria

*__Correspondence__

brigitte.kircher@i-med.ac.at

Table of Content

1. Characterisation of the ligands L1 – L3 ..... S3
1.1. ^{1}H NMR spectra of the ligands S3
1.2. ^{13}C NMR spectra of the ligands S6
1.3. ^{1}H – ^{13}C heteronuclear single quantum correlation (HSQC) of the ligands S9
1.4. FT-IR spectra of the ligands S10
1.5. HPLC chromatograms of the ligands S12
2. Characterisation of the complexes X1 – X3 ..... S14
2.1. FT-IR spectra of the complexes S14
2.2. HPLC chromatograms of the complexes S16
2.3. NMR spectra with Evans method of the complexes S18
2.4. EPR spectra of the complexes S21
3. Reactivity toward biological nucleophiles S22
4. Biological activity S24

1. Characterisation of the ligands L1 – L3

1.1. ^{1}H NMR spectra of the ligands

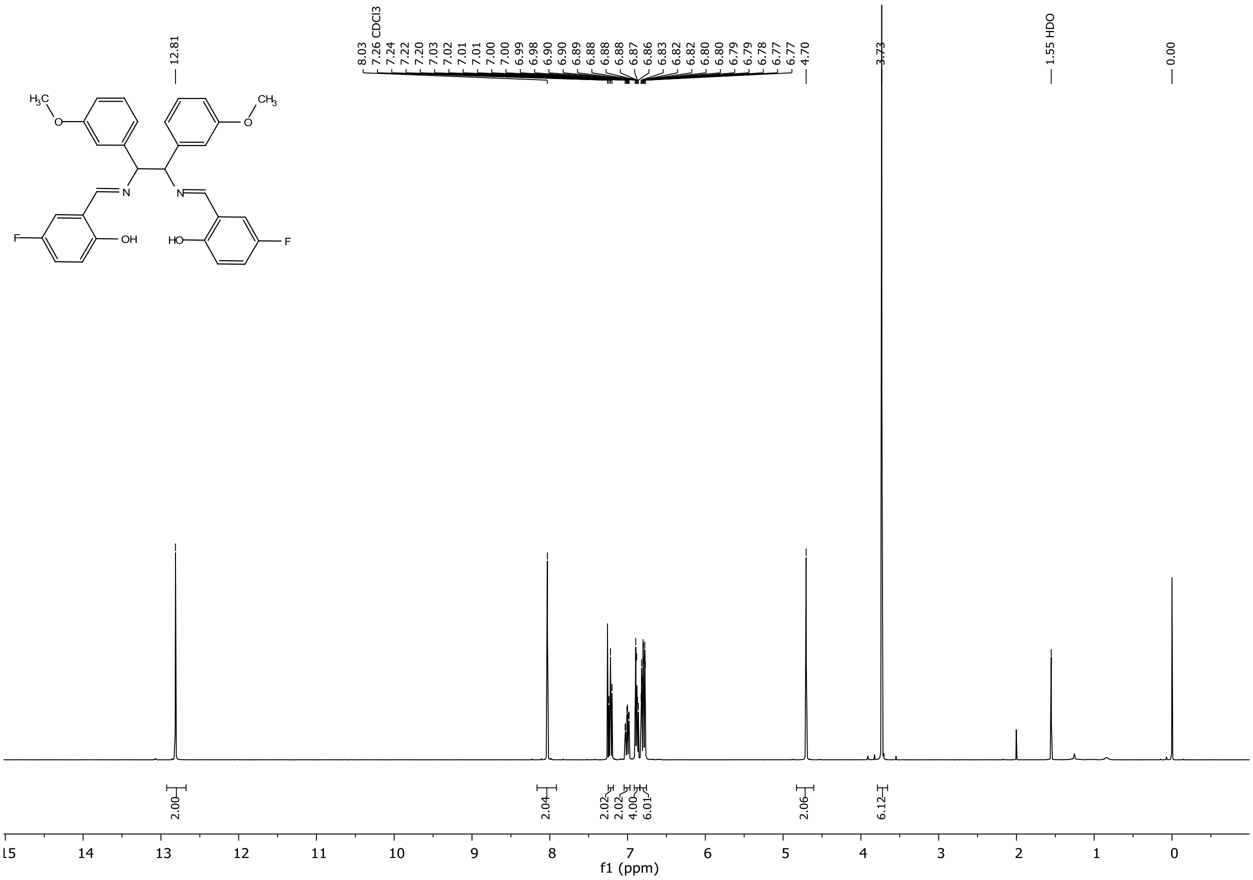

Figure S1: ^{1}H NMR of **L1** in $CDCl_3$

^{1}H NMR (400 MHz, $CDCl_3$-*d*) δ 12.81 (s, 2H), 8.03 (s, 2H), 7.22 (t, *J* = 7.9 Hz, 2H), 7.01 (ddd, *J* = 9.1, 8.1, 3.1 Hz, 2H), 6.92 – 6.84 (m, 4H), 6.84 – 6.75 (m, 6H), 4.70 (s, 2H), 3.73 (s, 6H)

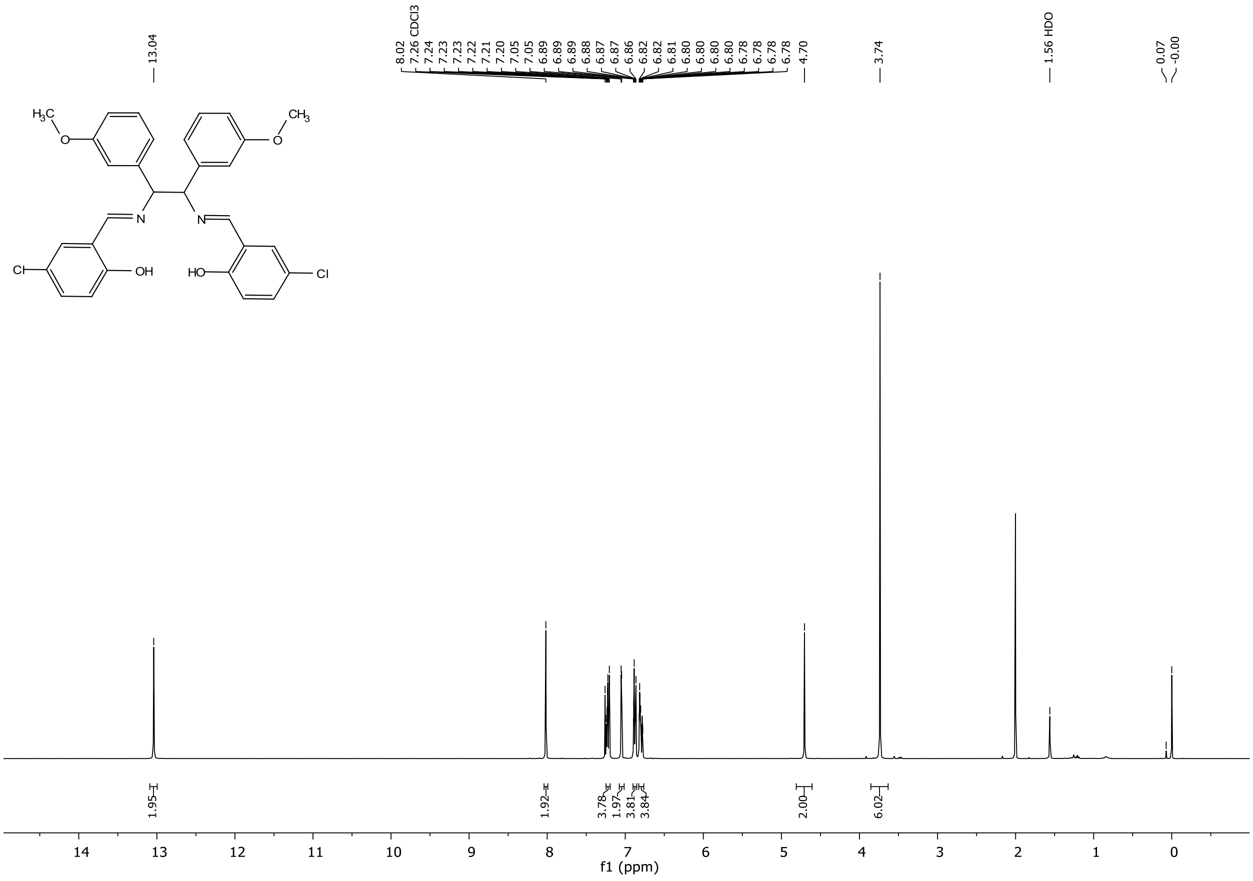

Figure S2: ^{1}H NMR of **L2** in $CDCl_3$

^{1}H NMR (400 MHz, $CDCl_3$-*d*) δ 13.04 (s, 2H), 8.02 (s, 2H), 7.27 – 7.18 (m, 4H), 7.05 (d, *J* = 2.5 Hz, 2H), 6.92 – 6.84 (m, 4H), 6.84 – 6.75 (m, 4H), 4.70 (s, 2H), 3.74 (s, 6H)

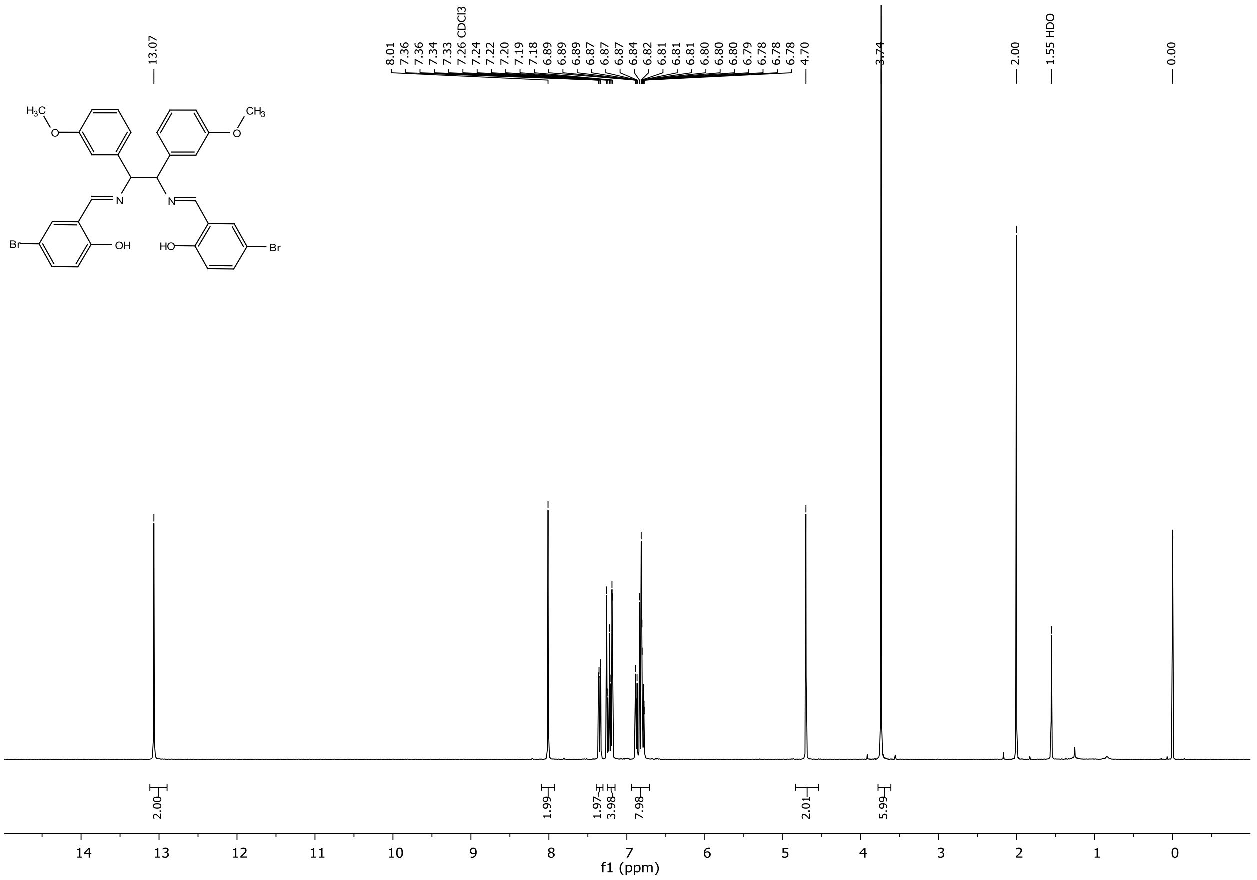

Figure S3: ^{1}H NMR of **L3** in $CDCl_3$

^{1}H NMR (400 MHz, $CDCl_3$-*d*) δ 13.07 (s, 2H), 8.01 (s, 2H), 7.35 (dd, *J* = 8.8, 2.5 Hz, 2H), 7.27 – 7.16 (m, 4H), 6.91 – 6.75 (m, 8H), 4.70 (s, 2H), 3.74 (s, 6H)

1.2. ^{13}C NMR spectra of the ligands

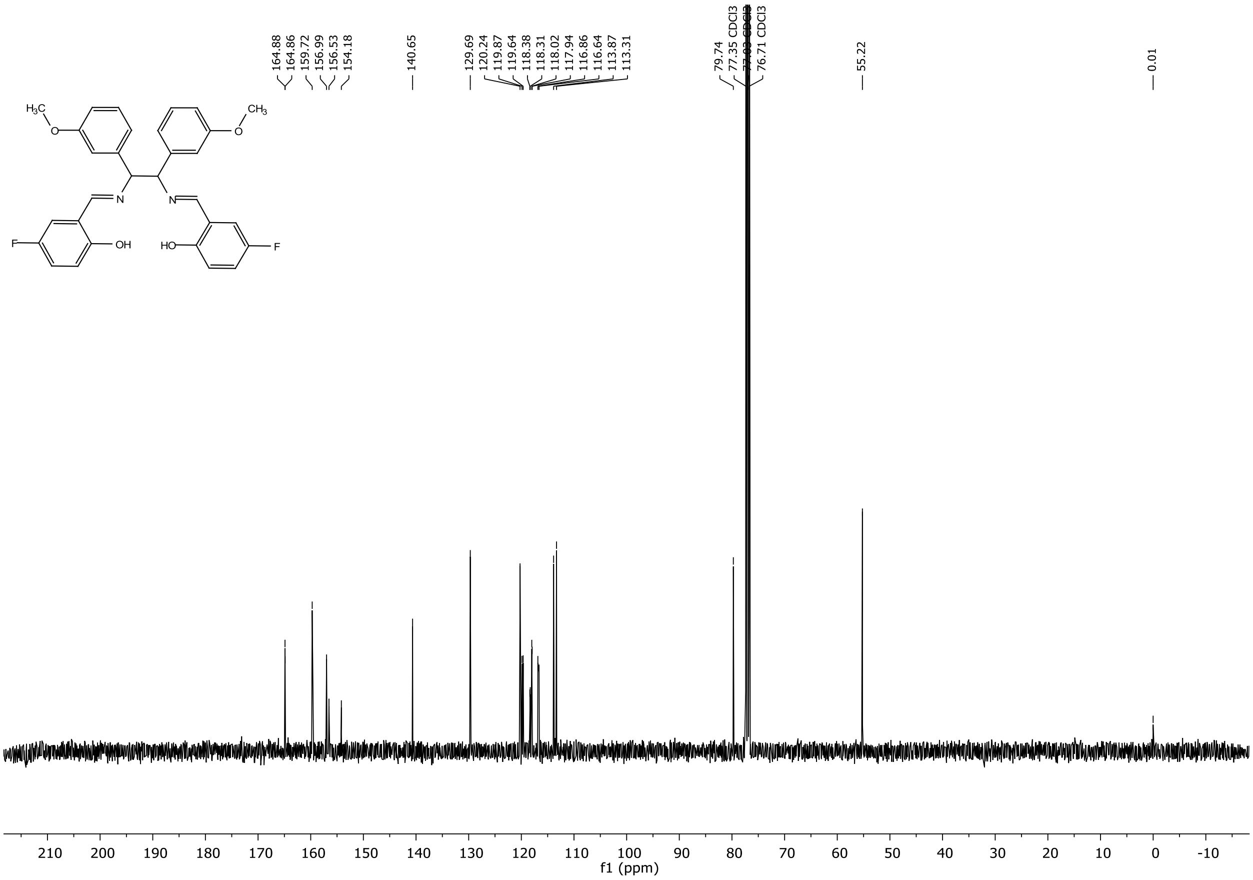

Figure S4: ^{13}C NMR of **L1** in $CDCl_3$

^{13}C NMR (101 MHz, $CDCl_3$-*d*) δ 164.87 (d, *J* = 2.7 Hz), 161.75 – 152.15 (m), 140.65, 129.69, 120.24, 119.76 (d, *J* = 23.2 Hz), 118.16 (dd, *J* = 37.0, 7.3 Hz), 116.75 (d, *J* = 22.8 Hz), 113.87, 113.31, 79.74, 55.22

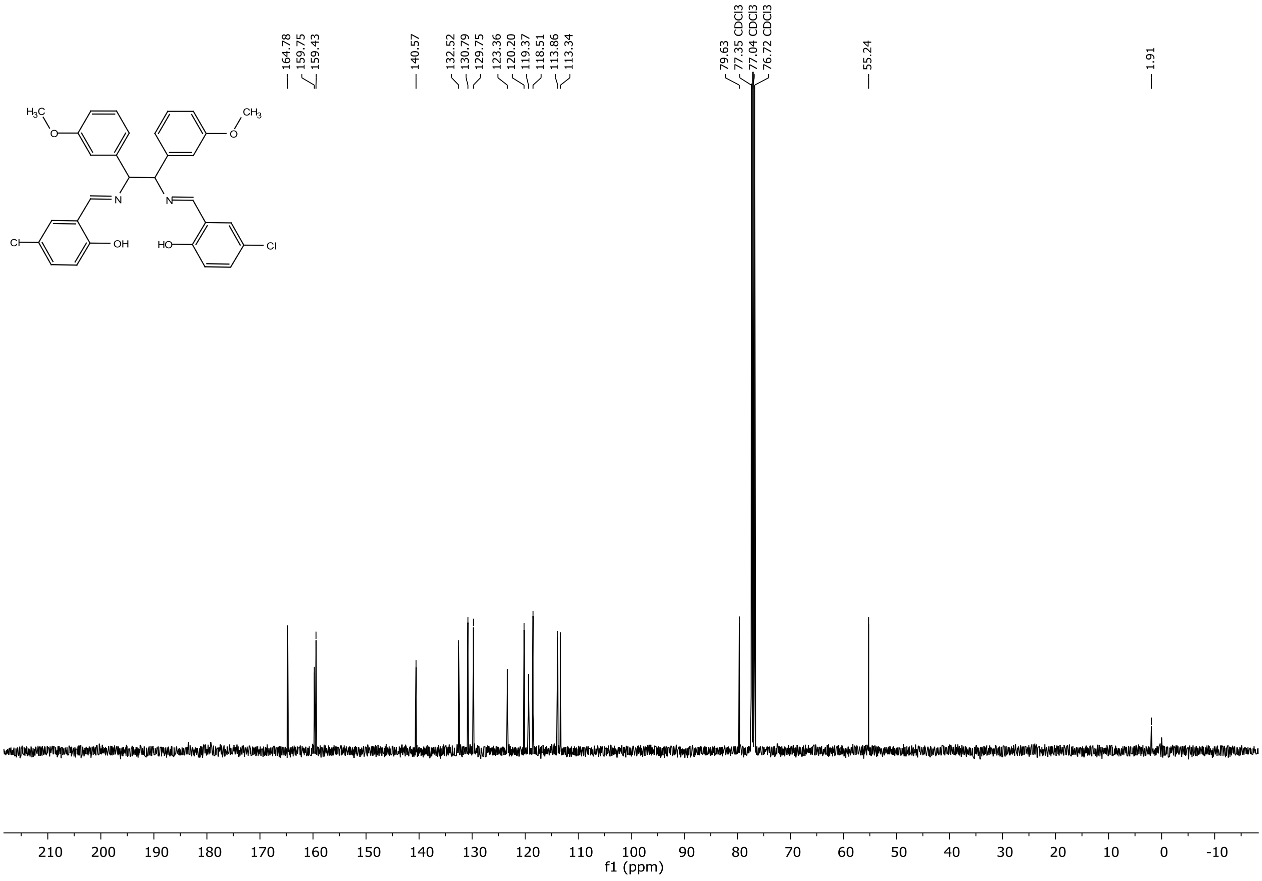

Figure S5: ^{13}C NMR of **L2** in $CDCl_3$

^{13}C NMR (101 MHz, $CDCl_3$-*d*) δ 164.78, 159.75, 159.43, 140.57, 132.52, 130.79, 129.75, 123.36, 120.20, 119.37, 118.51, 113.86, 113.34, 79.63, 55.24

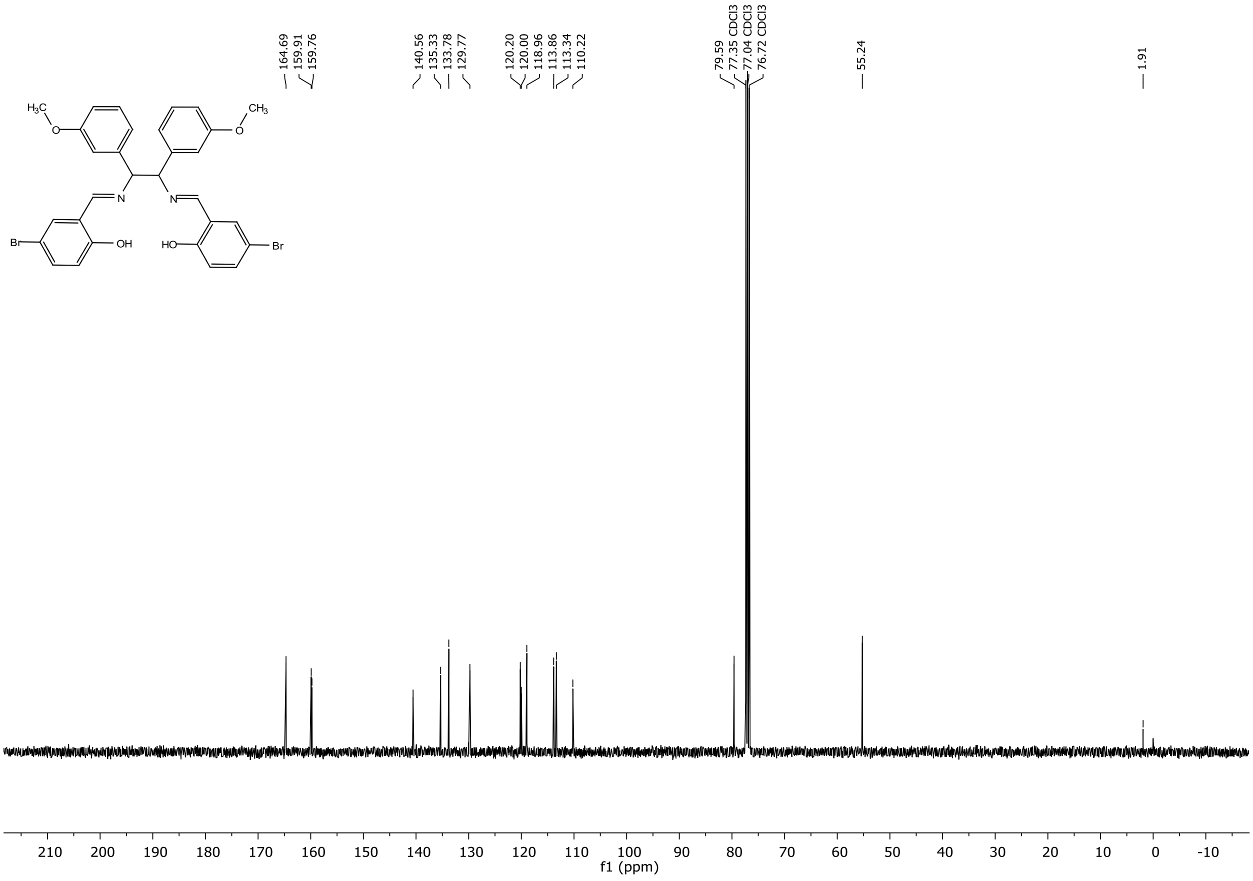

Figure S6: ^{13}C NMR of **L3** in $CDCl_3$

^{13}C NMR (101 MHz, $CDCl_3$-*d*) δ 164.69, 159.91, 159.76, 140.56, 135.33, 133.78, 129.77, 120.20, 120.00, 118.96, 113.86, 113.34, 110.22, 79.59, 55.24

1.3. ^{1}H – ^{13}C heteronuclear single quantum correlation (HSQC) of the ligands

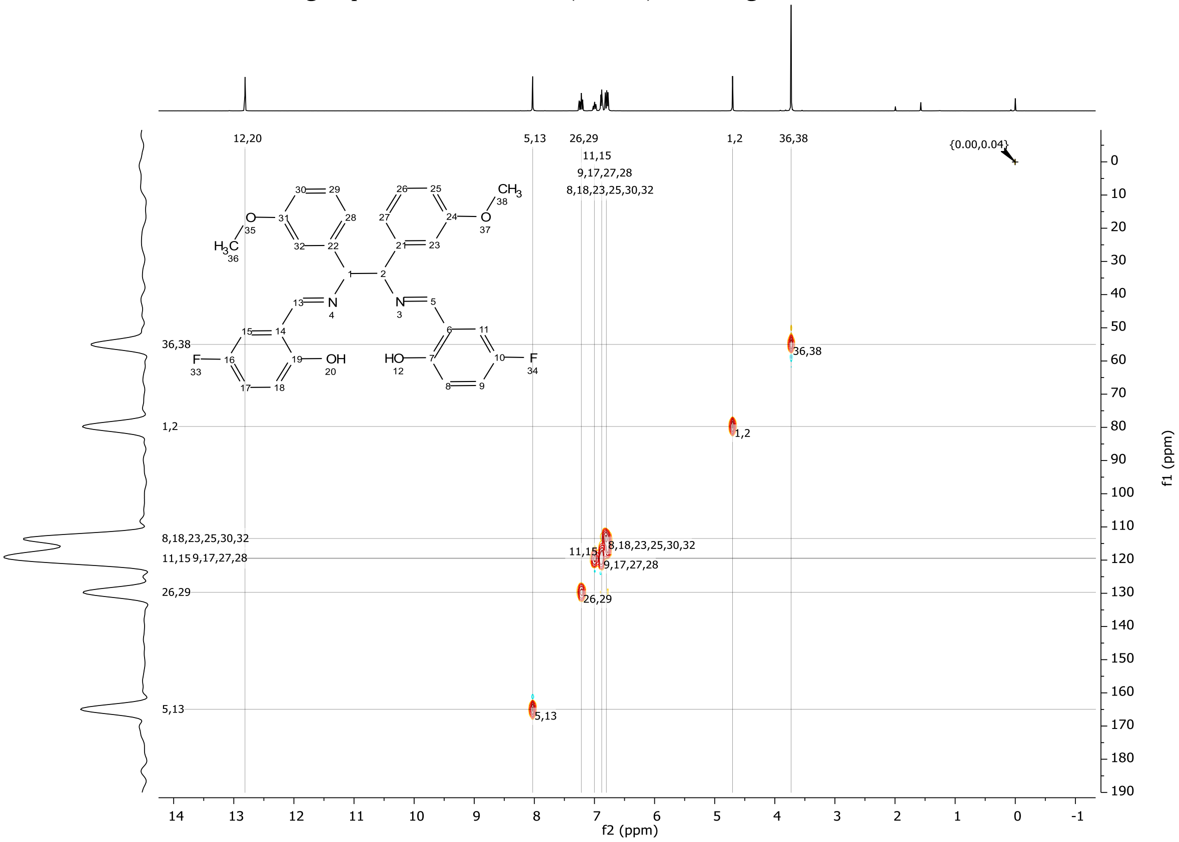

Figure S7: ^{1}H – ^{13}C HSQC spectrum of **L1** in $CDCl_3$

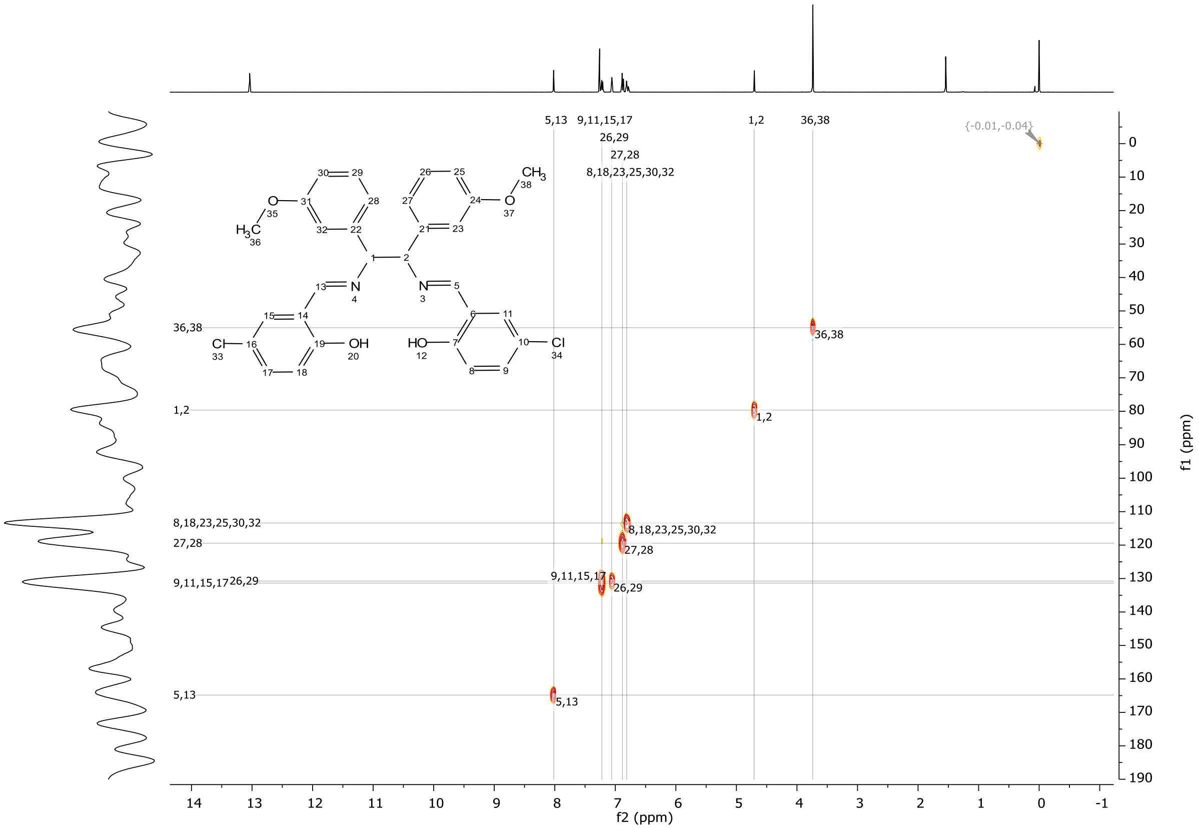

Figure S8: ^{1}H – ^{13}C HSQC spectrum of **L2** in $CDCl_3$

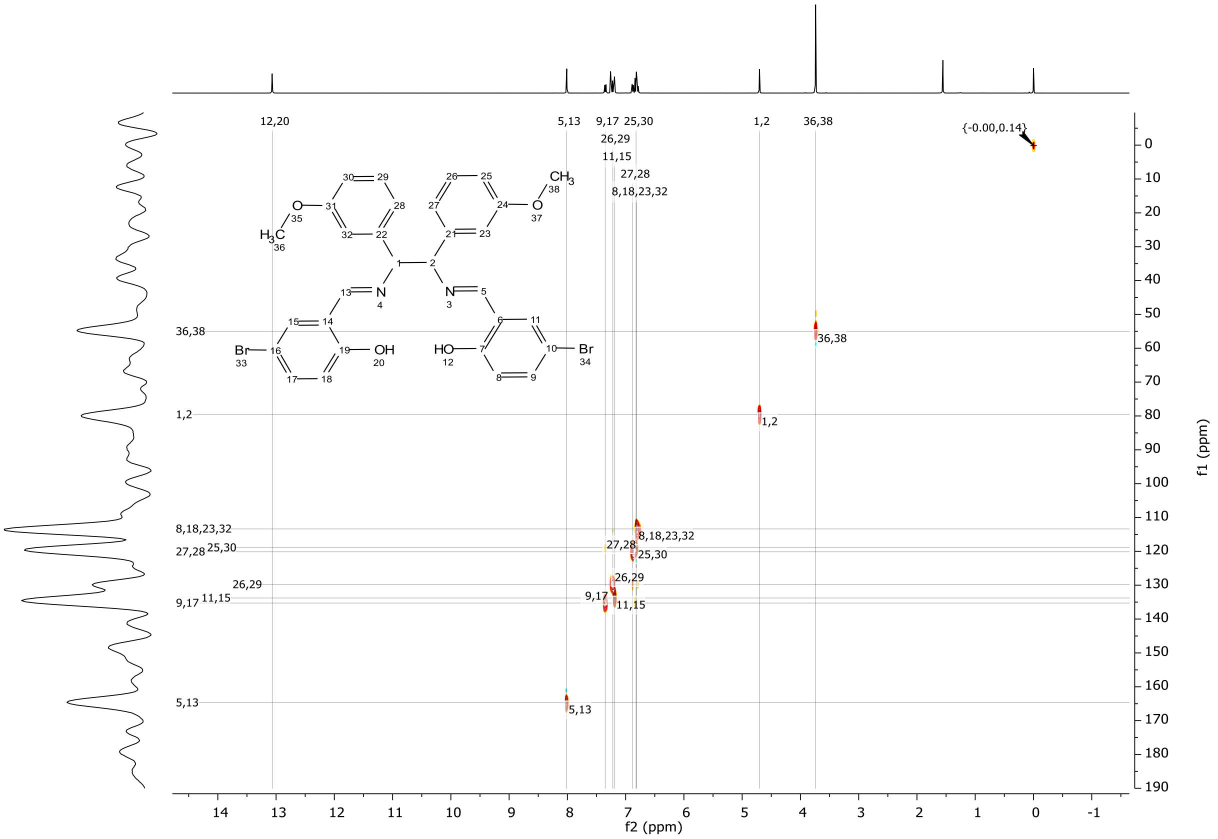

Figure S9: ^{1}H – ^{13}C HSQC spectrum of **L3** in $CDCl_3$

1.4. FT-IR spectra of the ligands

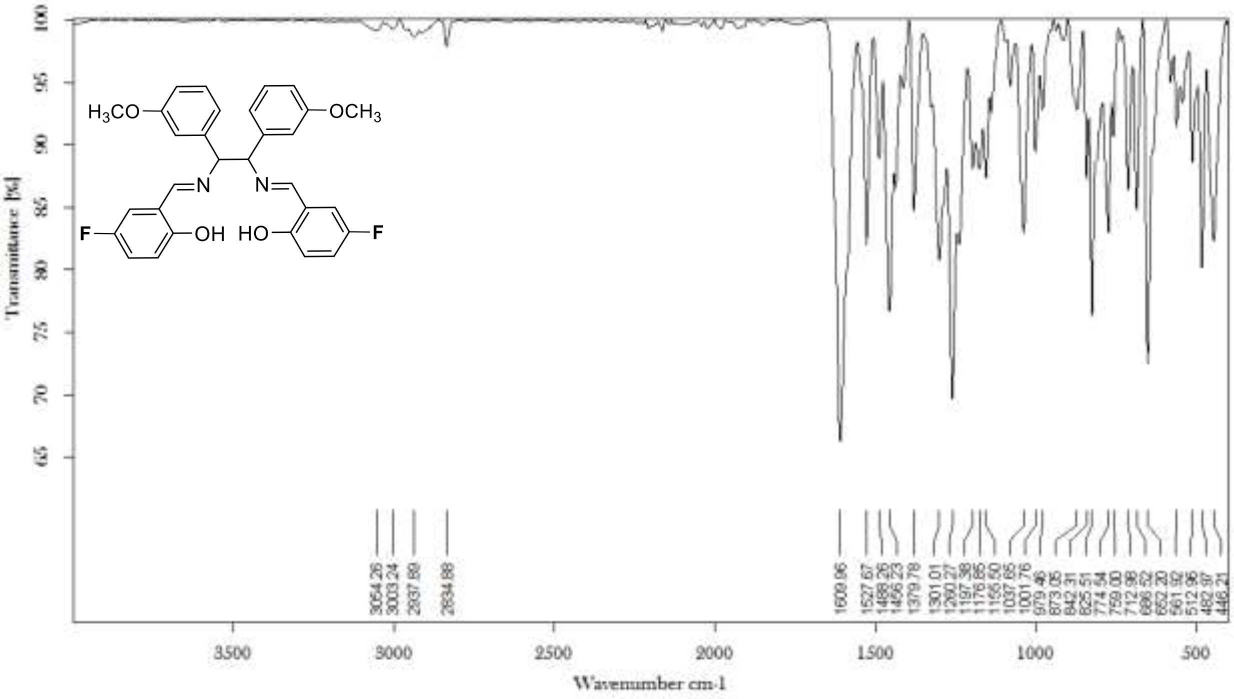

Figure S10: FT-IR spectrum of the neat **L1**

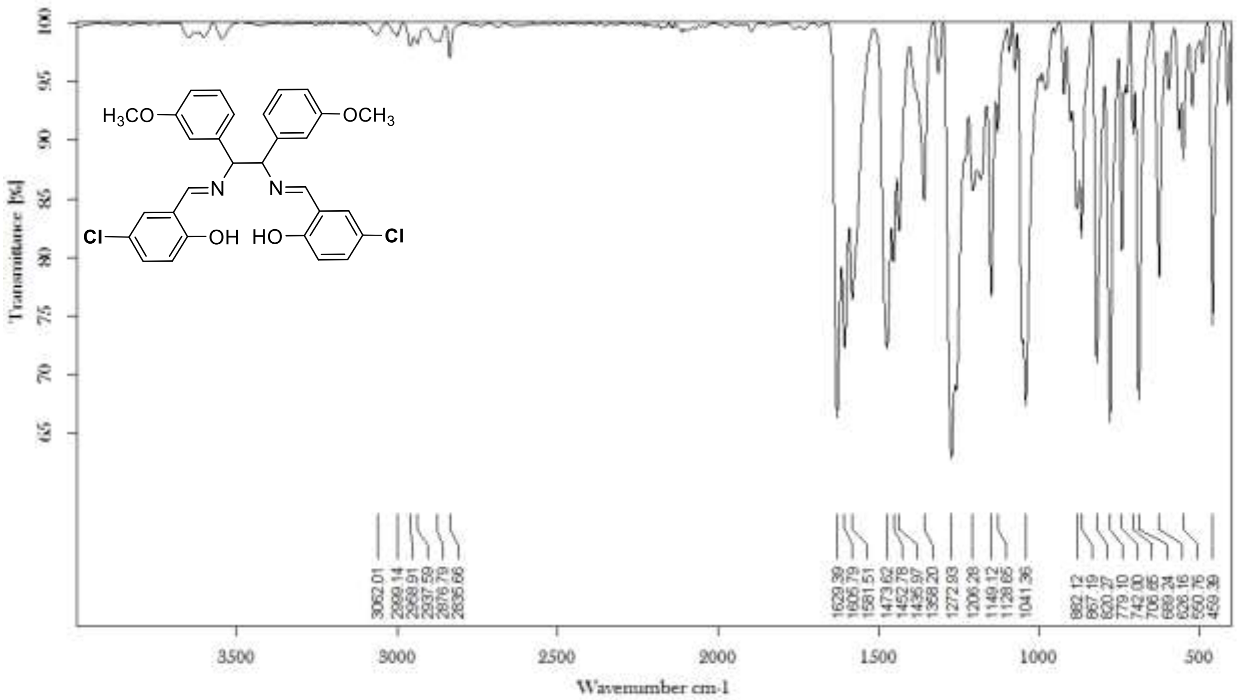

Figure S11: FT-IR spectrum of the neat **L2**

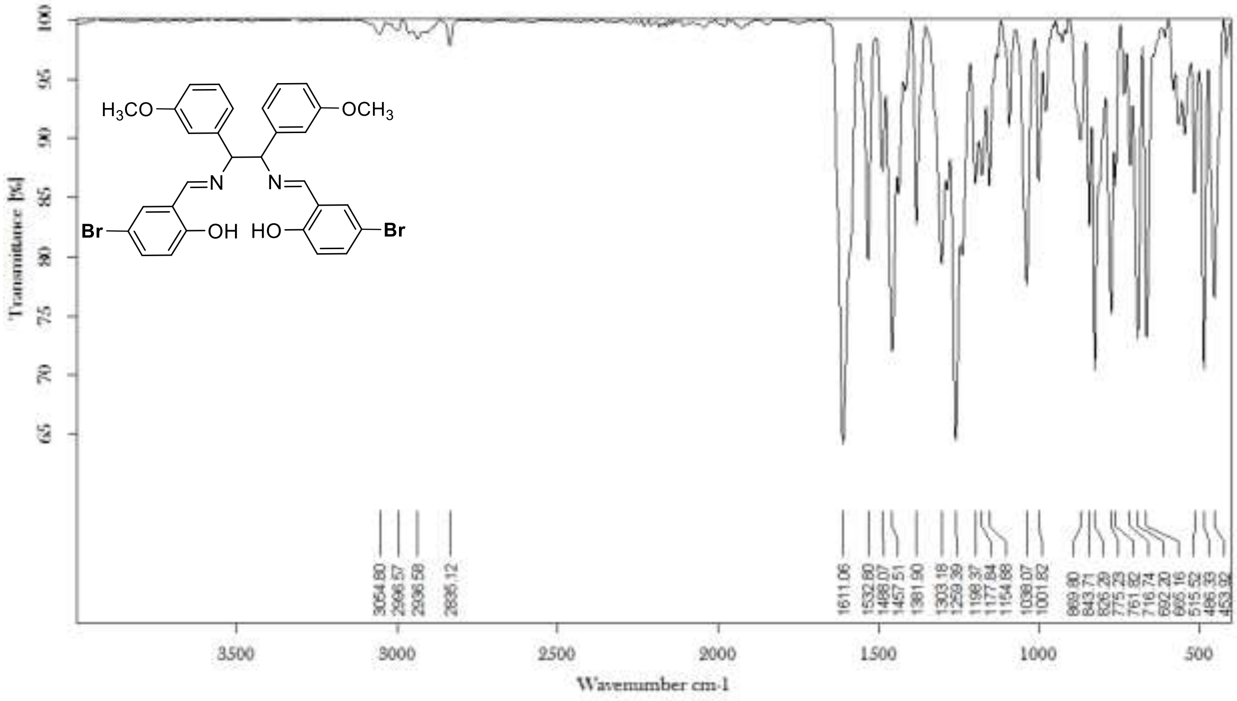

Figure S12: FT-IR spectrum of the neat **L3**

1.5. HPLC chromatograms of the ligands

Table S1: HPLC data **L1** – **L3**

Nr.	Ret. Time in Min.	Area %
**L1**	2.865	96.378
**L2**	4.713	95.724
**L3**	5.536	95.852

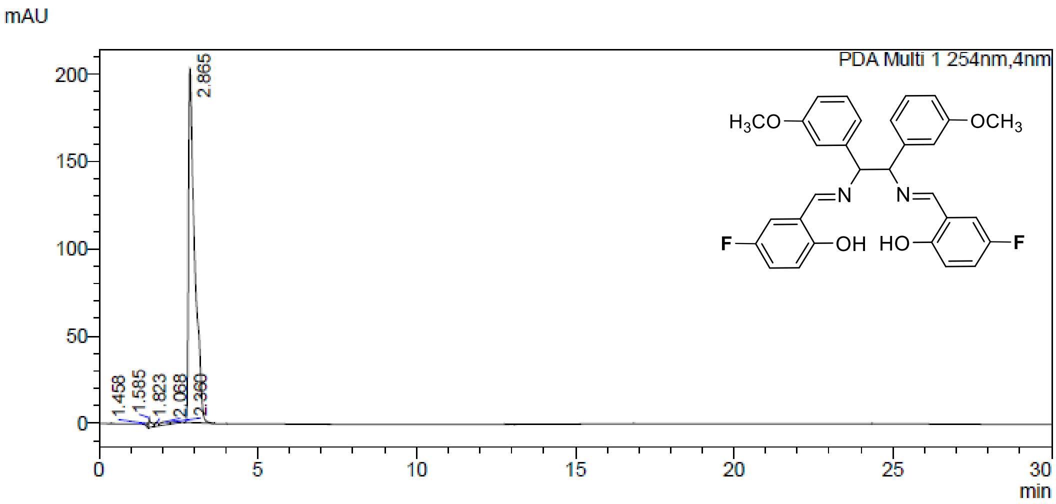

Figure S13: HPLC chromatogram of **L1**

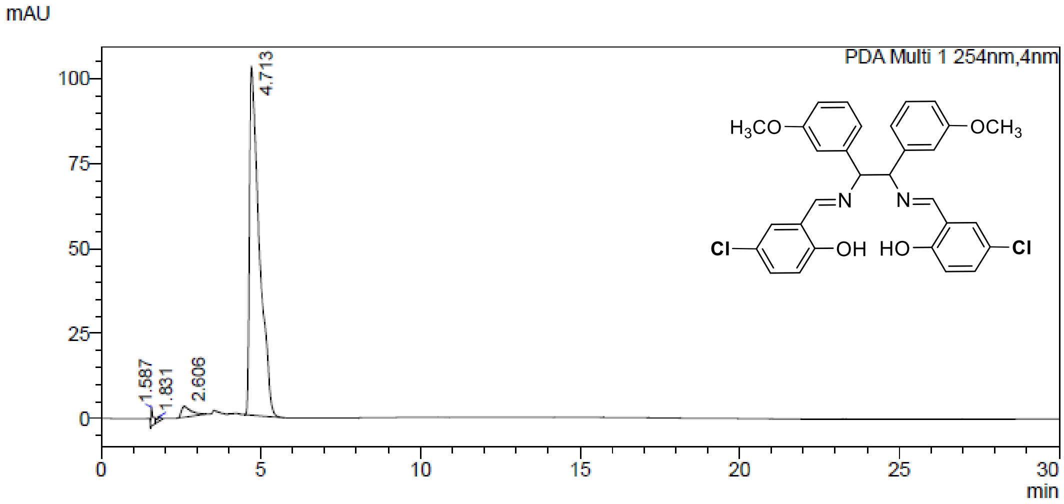

Figure S14: HPLC chromatogram of **L2**

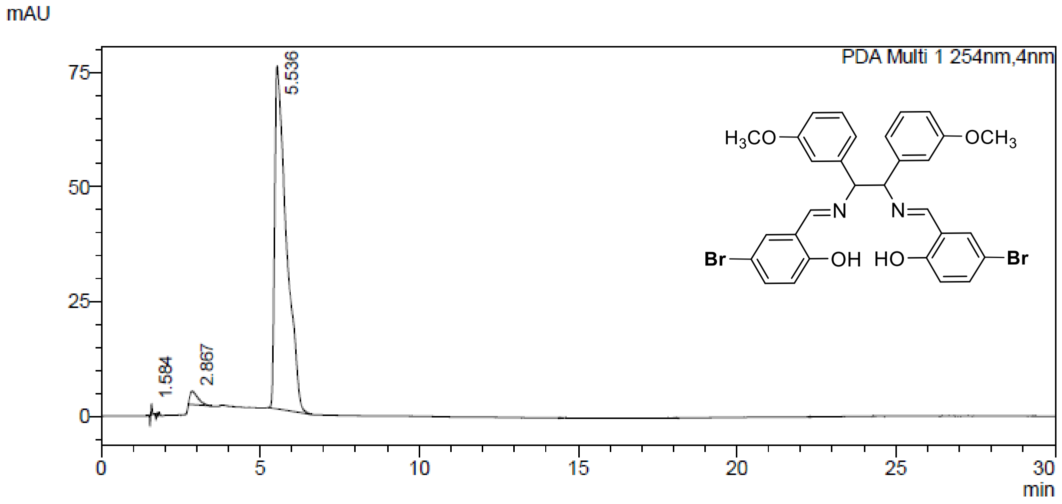

Figure S15: HPLC chromatogram of **L3**

2. Characterisation of the complexes X1 – X3

2.1. FT-IR spectra of the complexes

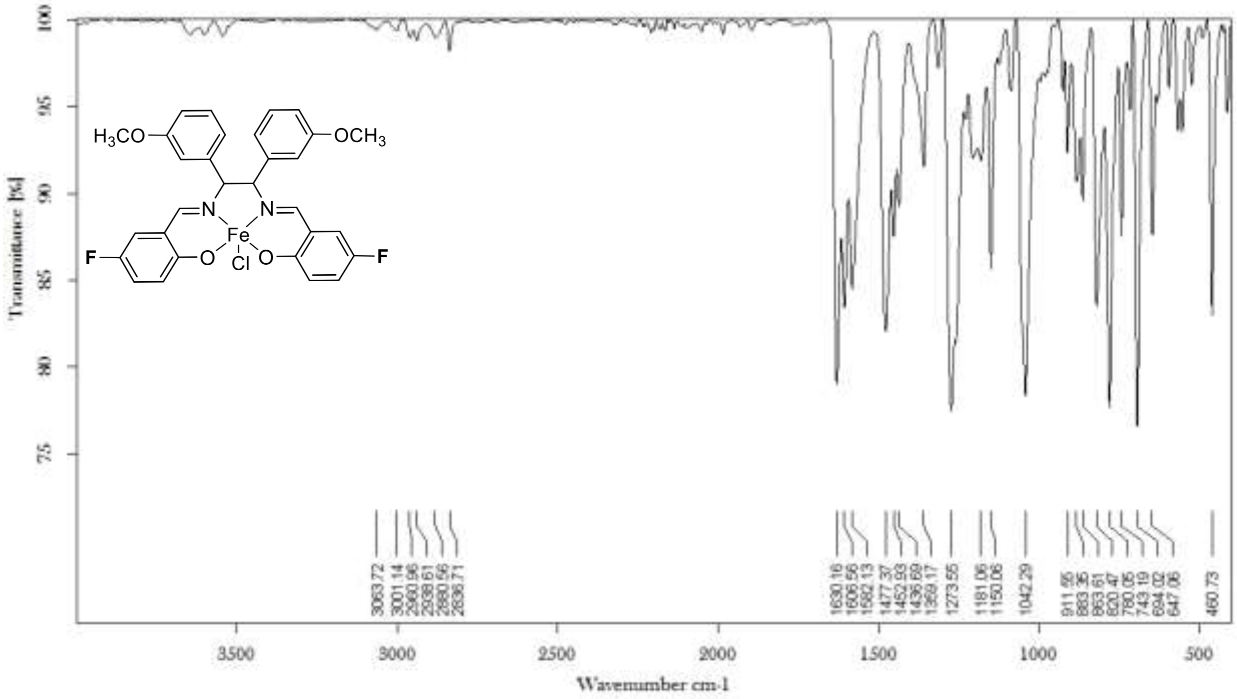

Figure S16: FT-IR spectrum of the neat **X1**

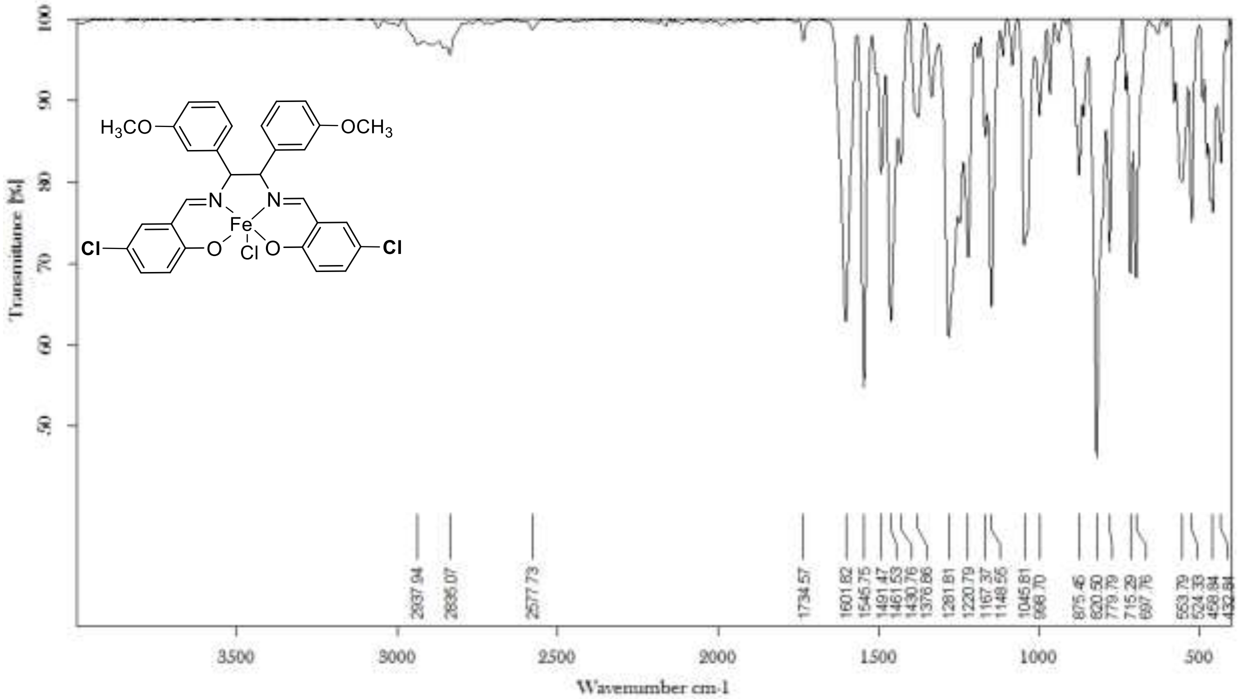

Figure S17: FT-IR spectrum of the neat **X2**

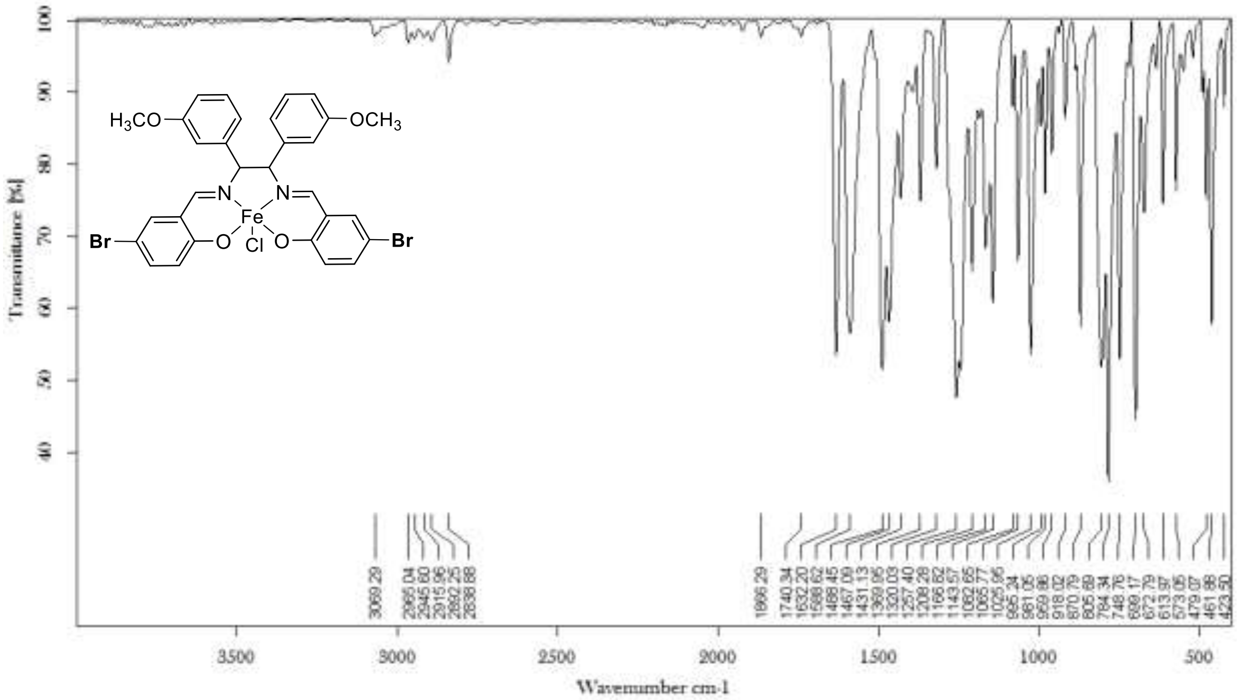

Figure S18: FT-IR spectrum of the neat **X3**

2.2. HPLC chromatograms of the complexes

Table S2: HPLC data **X1** – **X3**

Nr.	Ret. Time in Min.	Area %
**X1**	4.291	99.933
**X2**	7.614	99.794
**X3**	9.634	99.425

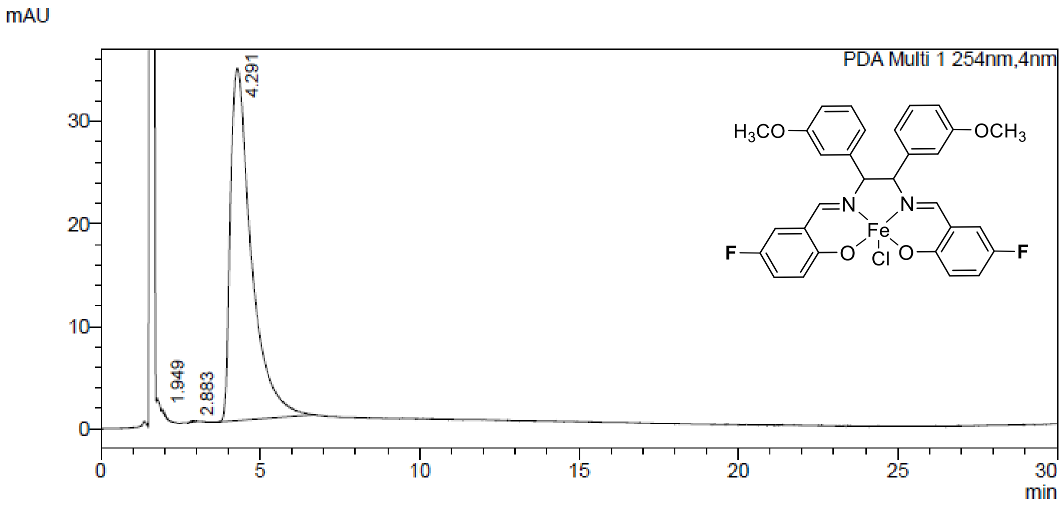

Figure S19: HPLC chromatogram of **X1**

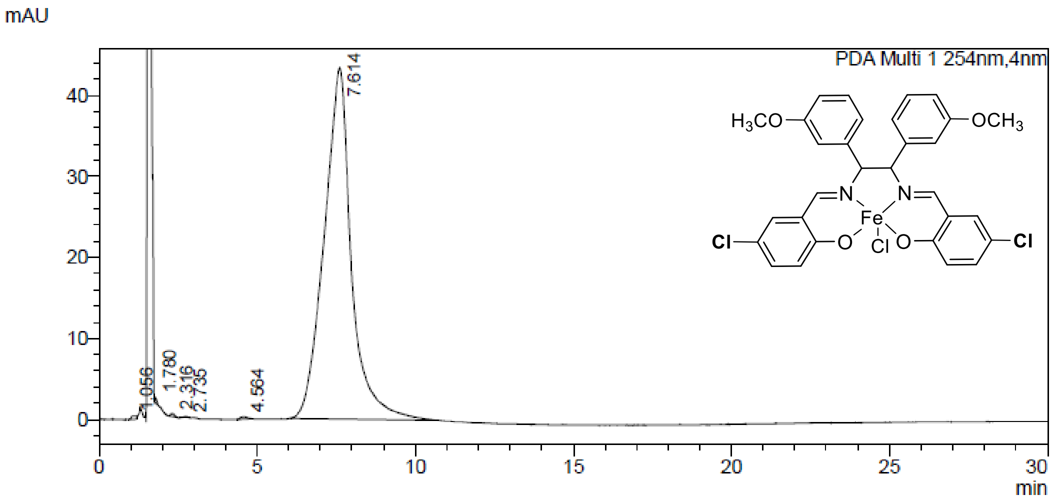

Figure S20: HPLC chromatogram of **X2**

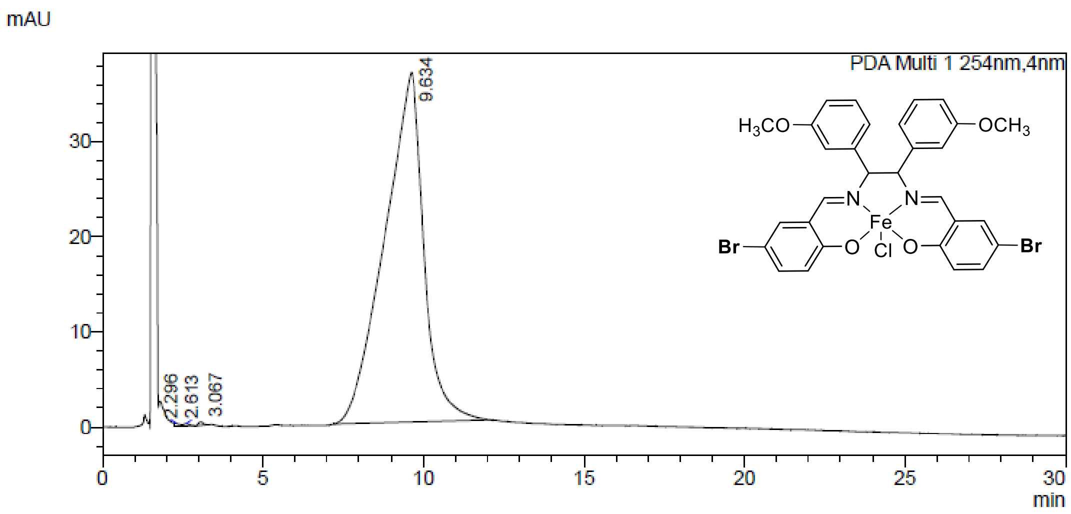

Figure S21: HPLC chromatogram of **X3**

2.3. NMR spectra with Evans method of the complexes

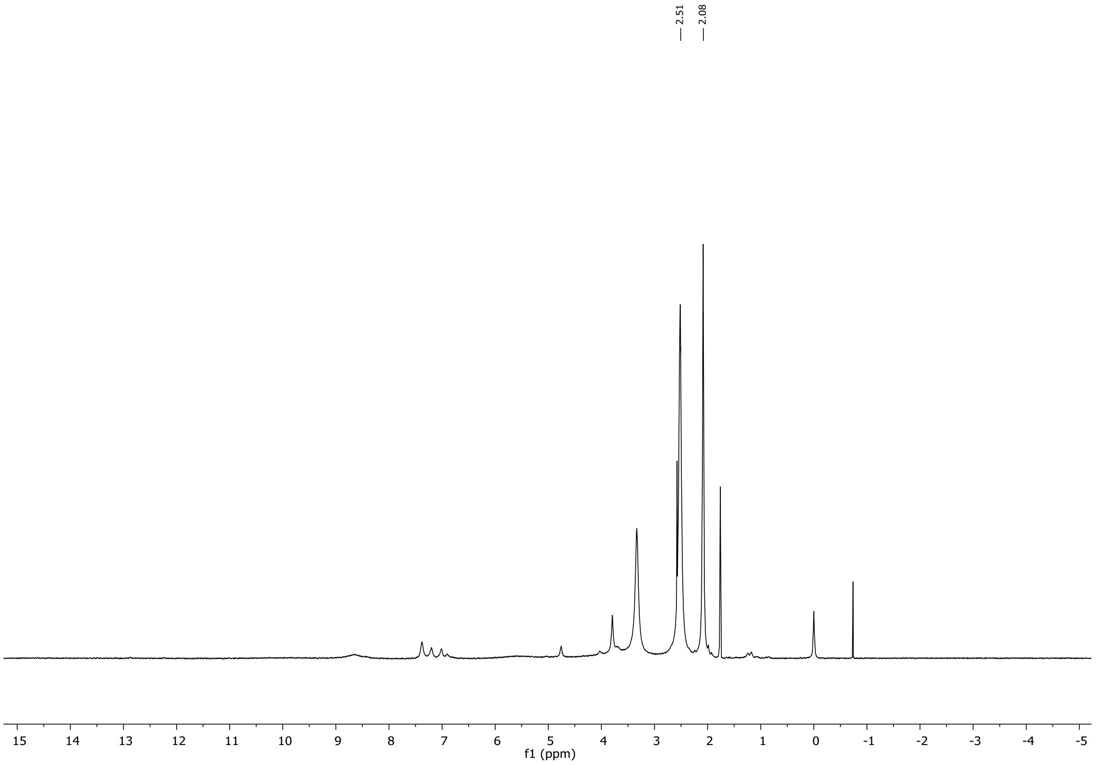

Figure S22: ^{1}H NMR spectrum of 5 mg of paramagnetic **X1** in DMSO-d_6 at 298 K with a DMSO-d_6 capillary. The shifted and unshifted solvent peaks are highlighted.

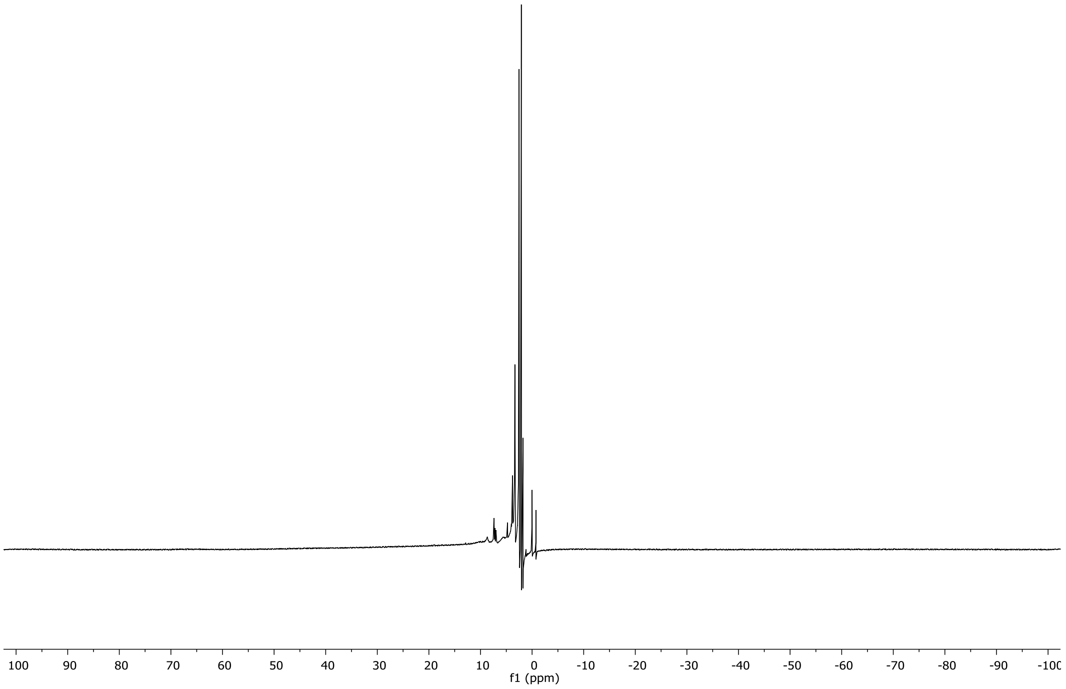

Figure S23: ^{1}H NMR spectrum of 5 mg of paramagnetic **X1** in DMSO-d_6 at 298 K

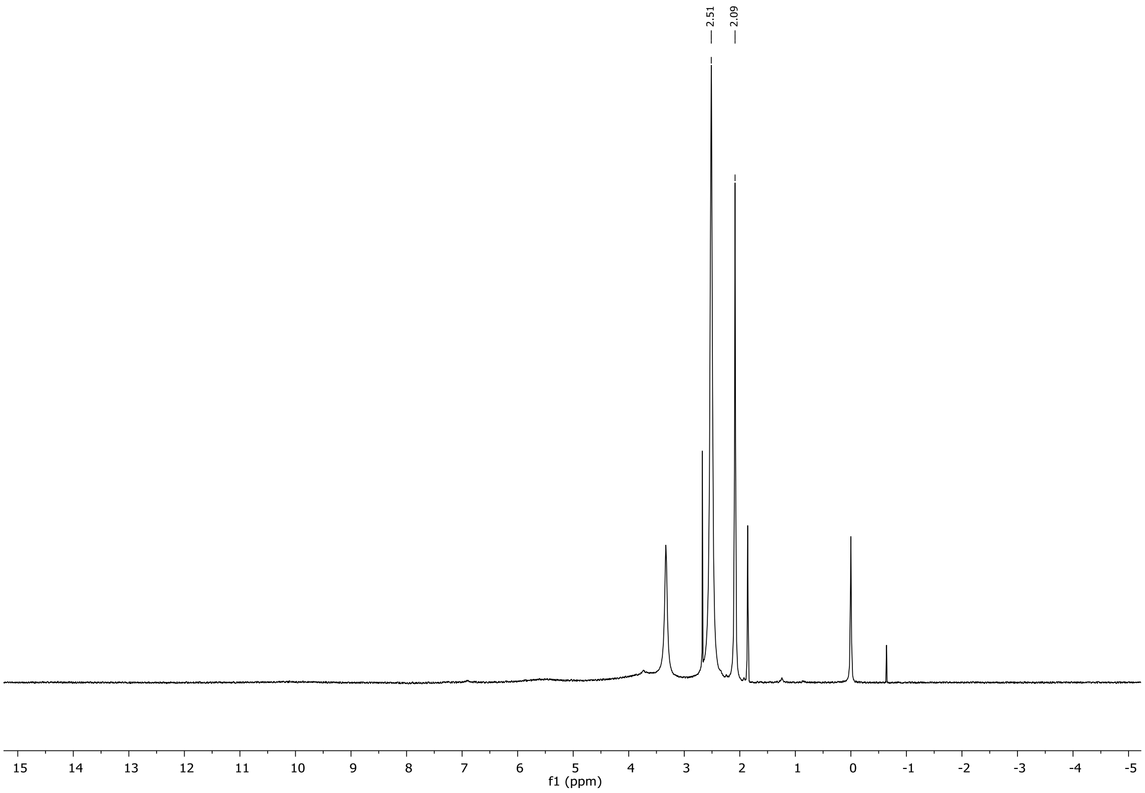

Figure S24: ^{1}H NMR spectrum of 5 mg of paramagnetic **X2** in DMSO-d_6 at 298 K with a DMSO-d_6 capillary. The shifted and unshifted solvent peaks are highlighted.

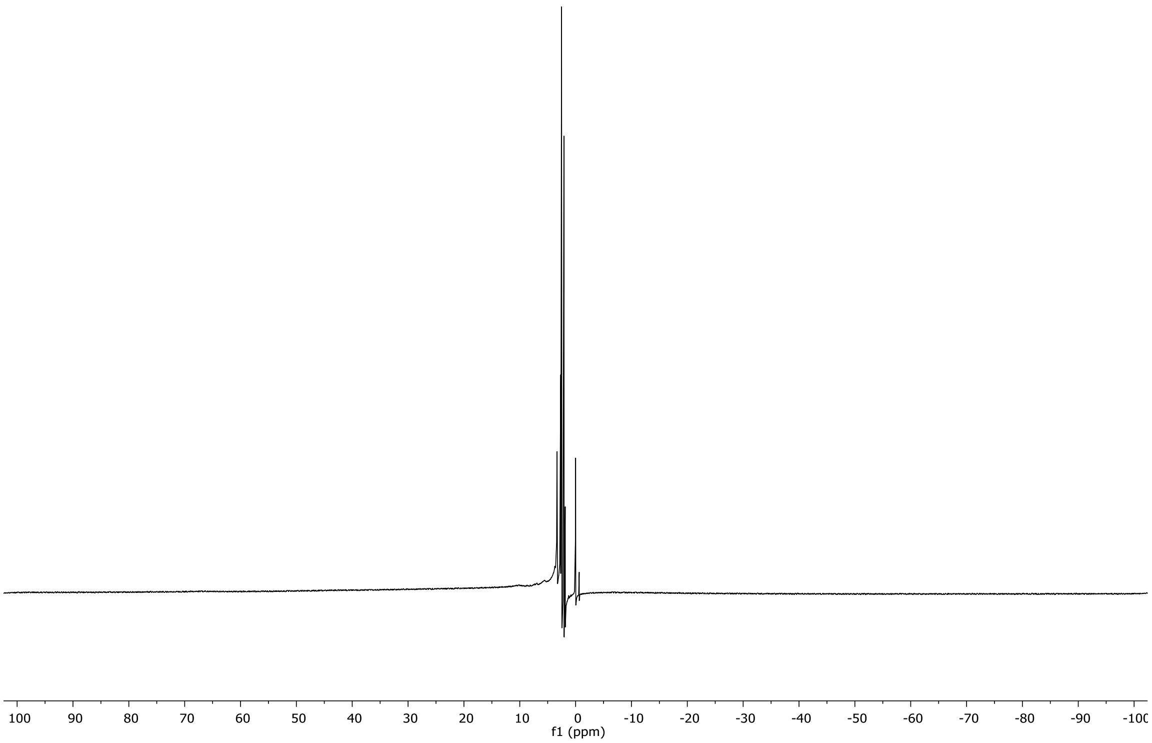

Figure S25: ^{1}H NMR spectrum of 5 mg of paramagnetic **X2** in DMSO-d_6 at 298 K

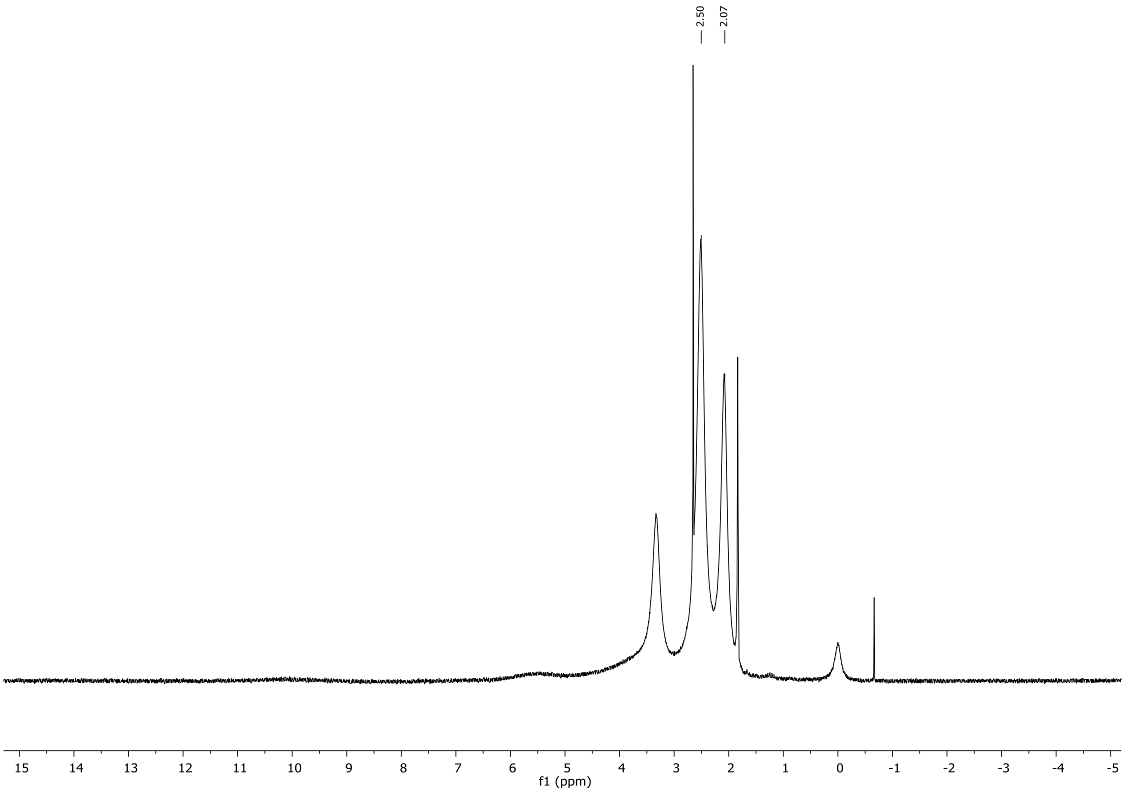

Figure S26: ^{1}H NMR spectrum of 5 mg of paramagnetic **X3** in DMSO-d_6 at 298 K with a DMSO-d_6 capillary. The shifted and unshifted solvent peaks are highlighted.

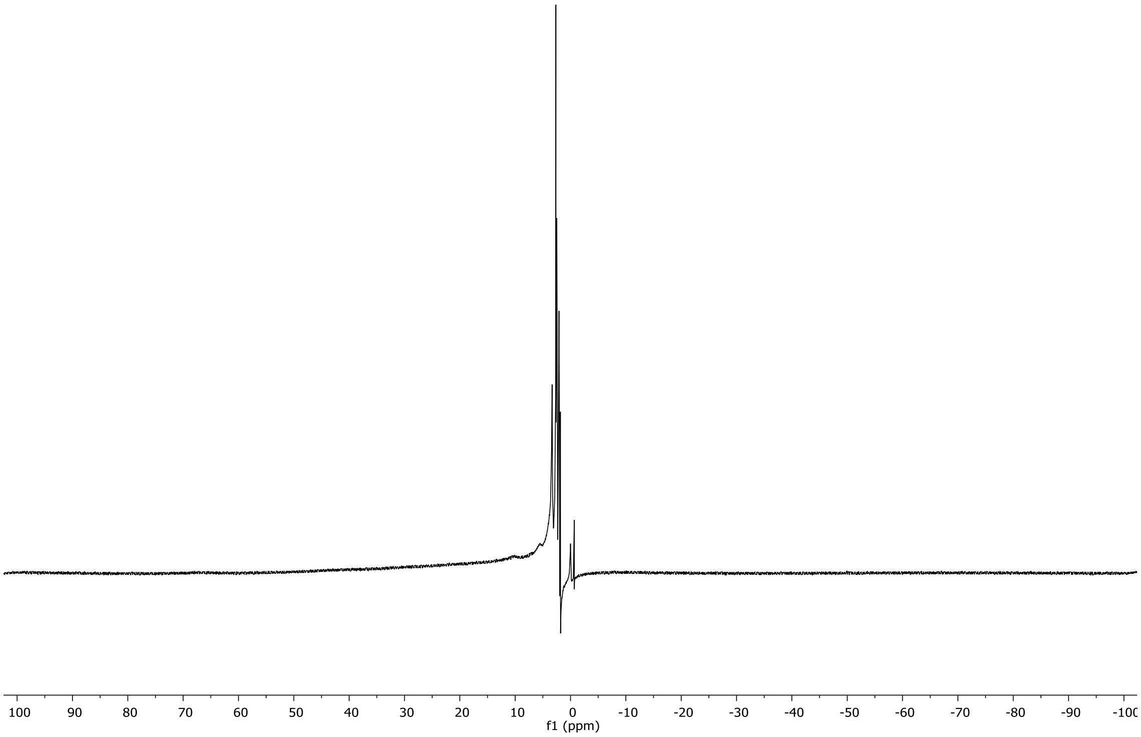

Figure S27: ^{1}H NMR spectrum of 5 mg of paramagnetic **X3** in DMSO-d_6 at 298 K

2.4. EPR spectra of the complexes

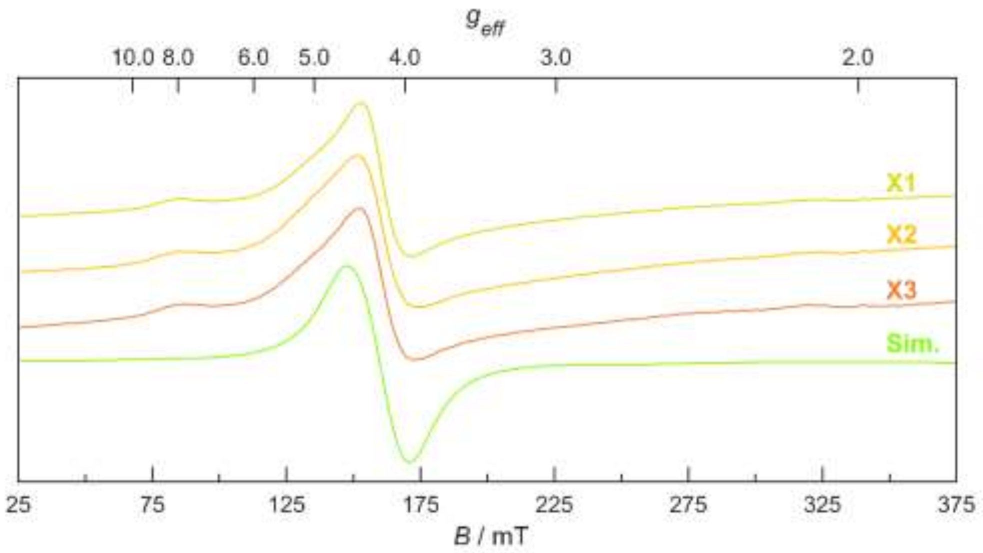

Figure S28: EPR spectra of **X1**, **X2** and **X3** in DMSO at 98 K. Simulation of a rhombic spin 5/2 Fe^{3+} system showing the observed signature. Simulation parameters: g_x=g_y=g_z=2.0023, D = 10 000 MHz, E/D = 0.28. Note: The intensity of the signal at g~7.9 is not accurately reproduced in the simulation due to line broadening effects.

3. Reactivity toward biological nucleophiles

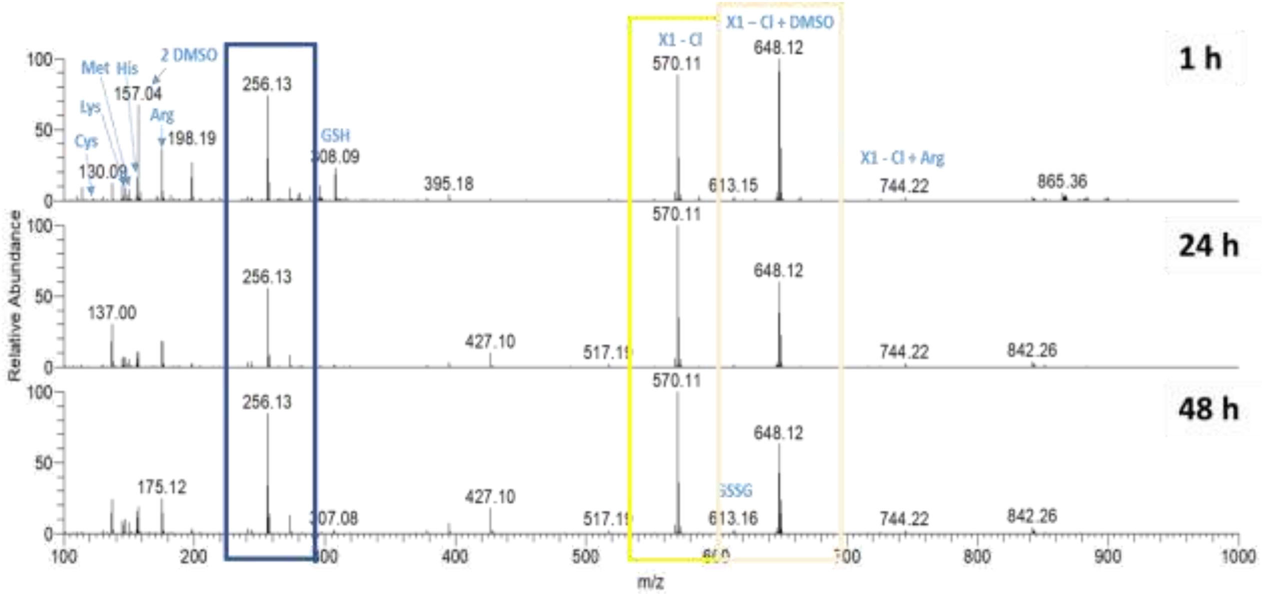

Figure S29: HR-MS spectra of **X1** in an aqueous solution containing equimolar amounts of Arg, Cys, His, Lys, Met and GSH.

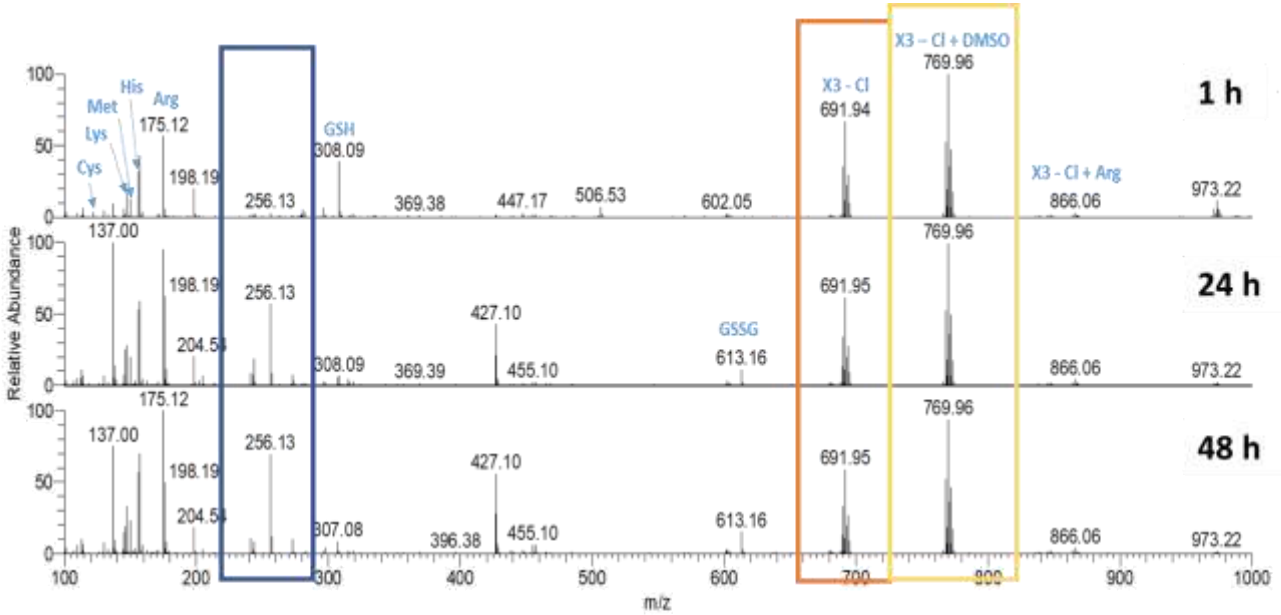

Figure S30: HR-MS spectra of **X3** in an aqueous solution containing equimolar amounts of Arg, Cys, His, Lys, Met and GSH.

Table S3: List of ions observed in the HR-MS analysis of **X1** – **X3** in aqueous solution. Oleamide is a common contaminant.

	species	$m_{exp.}$	m_{calc}	error [ppm]
**biomolecules**	$[Cys + H]^+$	122.0272	122.0270	1.64
	$[Lys + H]^+$	147.1130	147.1128	1.36
	$[Met + H]^+$	150.0585	150.0583	1.33
	$[His + H]^+$	156.0769	156.0768	0.64
	$[Arg + H]^+$	175.1191	175.1190	0.57
	$[GSH + H]^+$	308.0914	308.0911	0.97
	$[GSSG + H]^+$	613.1604	613.1592	1.96
**hydrolysis products**	$[C_{16}H_{17}O_2N + H]^+$	256.1336	256.1332	1.56
	$[C_{16}H_{20}O_2N_2 + H]^+$	273.1602	273.1598	1.46
	$[C_{23}H_{20}O_3N_1Cl + H]^+$	394.1212	394.1204	2.03
	$[C_{23}H_{23}O_3N_2Cl + H]^+$	411.1478	411.1470	1.95
**X1**	$[X1 - Fe - Cl + 3H]^+$	517.1943	517.1933	1.93
	$[X1 - Cl]^+$	570.1057	570.1049	1.40
	$[X1 - Cl + DMSO]^+$	648.1198	648.1190	1.23
	$[X1 - Cl + Lys]^+$	716.2118	716.2104	1.95
	$[X1 - Cl + His]^+$	725.1758	725.1741	2.34
	$[X1 - Cl + Arg]^+$	744.2180	744.2167	1.75
	$[X1 - Cl + C_{16}H_{20}O_2N_2]^+$	842.2592	842.2573	2.26
	$[X1 - Cl + GSH]^+$	877.1903	877.1888	1.71
**X2**	$[X2 - Cl]^+$	602.0470	602.0457	2.16
	$[X2 - Cl + DMSO]^+$	680.0611	680.0596	2.21
	$[X2 - Cl + Lys]^+$	748.1531	748.1512	2.54
	$[X2 - Cl + His]^+$	757.1170	757.1152	2.38
	$[X2 - Cl + Arg]^+$	776.1594	776.1574	2.58
	$[X2 - Cl + oleamide]^+$	883.3197	883.3174	2.60
	$[X2 - Cl + GSH]^+$	909.1316	909.1295	2.31
**X3**	$X3 - Cl]^+$	691.9440	691.9426	2.02
	$[X3 - Cl + DMSO]^+$	769.9585	769.9566	2.47
	$[X3 - Cl + Lys]^+$	838.0500	838.0482	2.15
	$[X3 - Cl + His]^+$	847.0140	847.0121	2.24
	$[X3 - Cl + Arg]^+$	866.0563	866.0543	2.31
	$[X3 - Cl + oleamide]^+$	973.2168	973.2145	2.36
	$[X3 - Cl + GSH]^+$	999.0286	999.0264	2.20

4. Biological activity

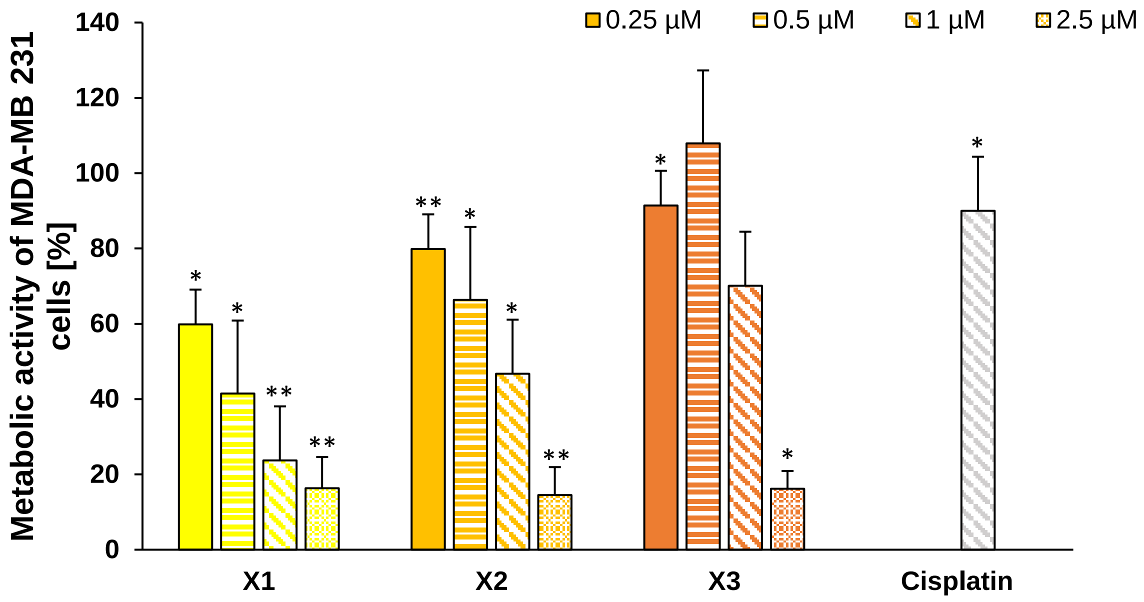

Figure S31: Effect of the iron(III) salene complexes **X1** – **X3** on the metabolic activity of MDA-MB 231. The mean proliferation + SE of eight independent experiments is plotted. The metabolic activity of cells only (without compound) was set at 100% (data not shown). The asterisks (* $p<0.05$, ** $p<0.005$ against no compound) represents statistical significance.

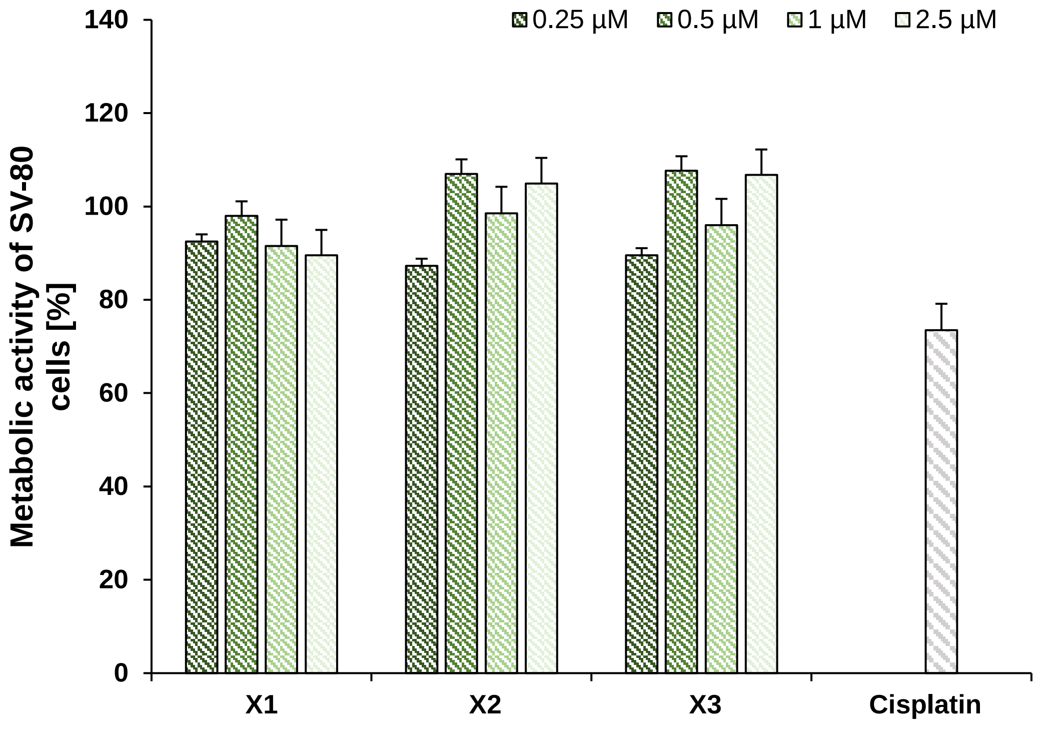

Figure S32: Effect of the iron(III) salene complexes **X1** – **X3** on the metabolic activity of SV-80 transformed fibroblasts. The mean metabolic activity + SE of five independent experiments is plotted. The metabolic activity of cells only (without compound) was set at 100%.

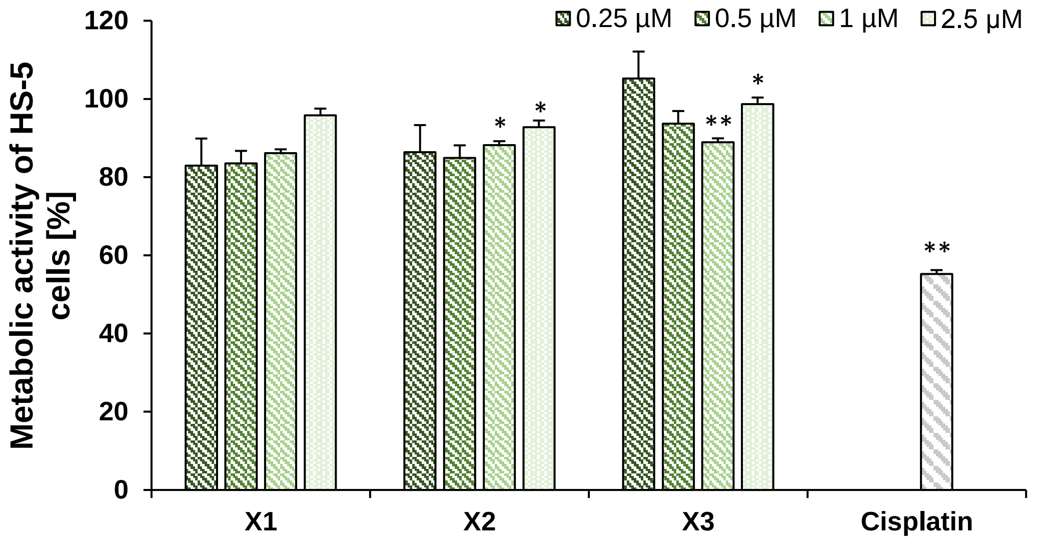

Figure S33: Effect of the iron(III) salene complexes **X1** – **X3** on the metabolic activity of HS-5 cells. The mean metabolic activity + SE of six independent experiments is plotted. The metabolic activity of cells only (without compound) was set at 100%. The asterisks (* $p<0.05$, ** $p<0.005$ against no compound) represents statistical significance.

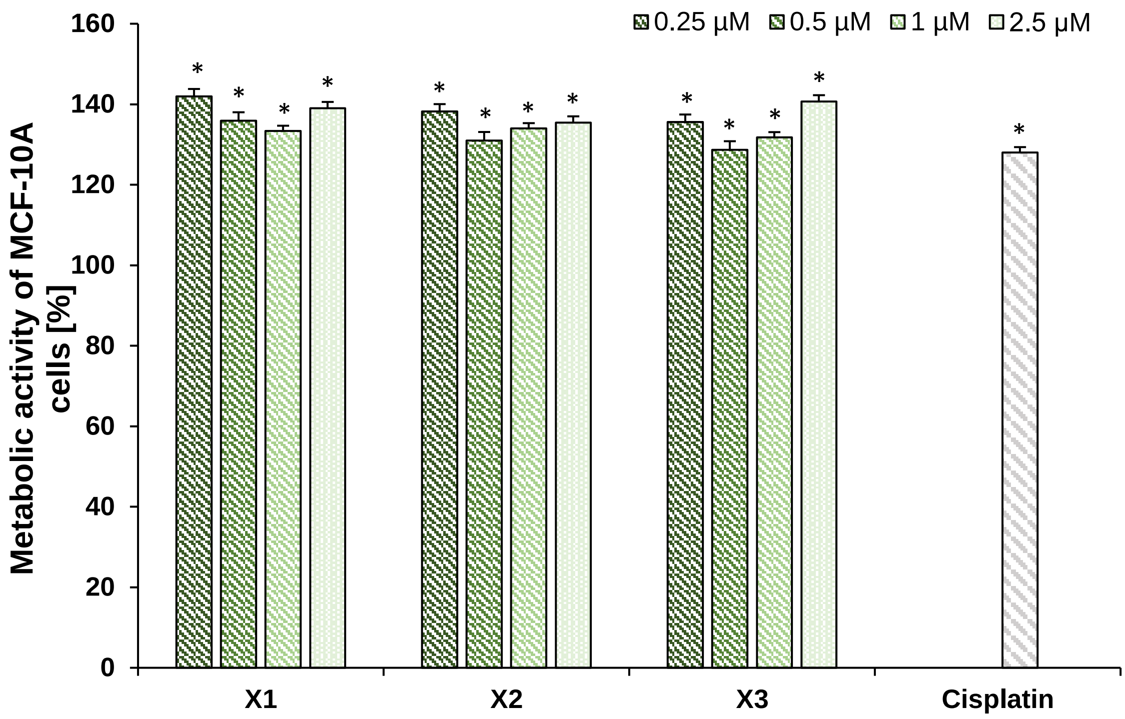

Figure S34: Effect of the iron(III) salene complexes **X1** – **X3** on the metabolic activity of MCF-10A cells. The mean metabolic activity + SE of six independent experiments is plotted. The metabolic activity of cells only (without compound) was set at 100%. The asterisks (* $p<0.005$ against no compound) represent statistical significance.
